# Deficient autophagy in epithelial stem cells drives aging in the freshwater cnidarian *Hydra*

**DOI:** 10.1242/dev.177840

**Published:** 2020-01-23

**Authors:** Szymon Tomczyk, Nenad Suknovic, Quentin Schenkelaars, Yvan Wenger, Kazadi Ekundayo, Wanda Buzgariu, Christoph Bauer, Kathleen Fischer, Steven Austad, Brigitte Galliot

**Affiliations:** 1 Department of Genetics and Evolution, Institute of Genetics and Genomics in Geneva (iGE3), University of Geneva, CH-1205 Geneva, Switzerland; 2 Department of Biology, University of Alabama at Birmingham, Birmingham, AL 35294, USA

**Keywords:** Aging model system, Autophagy sensor, Epithelial stem cells, *Hydra* regeneration, Evolution of aging, p62/SQSTM1, WIPI2, Rapamycin

## Abstract

*Hydra* possesses three distinct stem cell populations that continuously self-renew and prevent aging in *Hydra vulgaris*. However, sexual animals from the *H. oligactis* cold-sensitive strain *Ho_CS* develop an aging phenotype upon gametogenesis induction, initiated by the loss of interstitial stem cells. Animals stop regenerating, lose their active behaviors and die within 3 months. This phenotype is not observed in the cold-resistant strain *Ho_CR*. To dissect the mechanisms of *Hydra* aging, we compared the self-renewal of epithelial stem cells in these two strains and found it to be irreversibly reduced in aging *Ho_CS* but sustained in non-aging *Ho_CR*. We also identified a deficient autophagy in *Ho_CS* epithelial cells, with a constitutive deficiency in autophagosome formation as detected with the mCherry-eGFP-LC3A/B autophagy sensor, an inefficient response to starvation as evidenced by the accumulation of the autophagosome cargo protein p62/SQSTM1, and a poorly inducible autophagy flux upon proteasome inhibition. In the non-aging *H. vulgaris* animals, the blockade of autophagy by knocking down *WIPI2* suffices to induce aging. This study highlights the essential role of a dynamic autophagy flux to maintain epithelial stem cell renewal and prevent aging.

## Abstract (precis)

**Summary:** Lack of epithelial stem cell renewal and deficient epithelial autophagy are the major causes of aging in *Hydra oligactis*, whereas lowering autophagy efficiency in the non-aging *Hydra vulgaris* induces an aging phenotype.

## INTRODUCTION

**Fig. 1.**
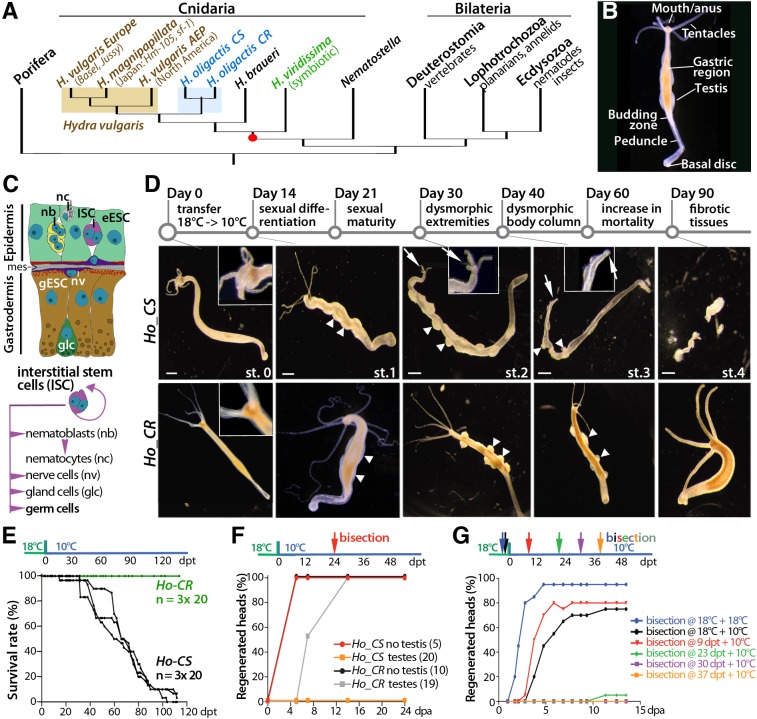
**Inducible aging phenotype in cold-sensitive *Hydra oligactis* (*Ho_CS*).** (A) Phylogenetic position of *Hydra* among metazoans. (B) Anatomy of a male *H. oligactis* animal. (C) Schematic view of *Hydra* gastric tissue. Mes, mesoglea. (D) Morphological changes observed in *Ho_CS* (top) and *Ho_CR* (bottom) animals at various time points after transfer to 10°C (day 0); arrowheads indicate testes, arrows indicate head regions. Scale bars: 500 µm. (E) Survival rates among *Ho_CR* and *Ho_CS* cohorts maintained at 10°C for 120 days. (F) Head regeneration in *Ho_CR* or *Ho_CS* animals selected for the presence or the absence of testes, bisected at mid-gastric level on day 24 post-transfer (dpt) and monitored for 24 days post-amputation (dpa). (G) Head regeneration measured in *Ho_CS* animals bisected at 18°C (blue, black) or at various time points after transfer to 10°C (9, 23, 30, 37 dpt) and monitored for 15 days.

Studies using short-lived invertebrate organisms, such as the fruit fly or the nematode, have dramatically improved our understanding of aging (Helfand and Rogina, 2003; Longo and Finch, 2003; Olsen et al., 2006). However, these model systems have some drawbacks, such as developmental pausing under stress, implying that the life-prolonging interventions may work through mechanisms not present in humans (Austad, 2009). Additionally, except for the *Drosophila* gut, adult somatic tissues do not self-renew in flies and nematodes, whereas self-renewal is an essential component of homeostasis in humans. Finally, a significant proportion of human orthologous genes were lost in fly and nematodes as evidenced by their presence in cnidarians, a bilaterian sister group (Kortschak et al., 2003; Wenger and Galliot, 2013; Schenkelaars et al., 2018). Therefore, additional invertebrate models could be profitably developed to help discover novel genes, pathways and mechanisms relevant for human aging (Valenzano et al., 2017). Among those, *Hydra*, a small carnivorous freshwater cnidarian polyp with a radially organized bi-layered body plan, is equipped with three distinct adult stem cell populations that continuously self-renew whatever the age of the animal (Fig. 1A-C). This dynamic homeostasis supports the amazing ability to regenerate any missing part (Galliot, 2012). In laboratory conditions, the mortality of *Hydra vulgaris* (*Hv*) remains negligible over the years as well-fed animals remain asexual, reproducing by budding without showing replicative aging (Brien, 1953; Martínez, 1998; Schaible et al., 2015).

The multifunctional but unipotent epithelial stem cells from the epidermis and the gastrodermis (eESCs and gESCs, respectively) cycle every 3-4 days whereas the multipotent interstitial stem cells (ISCs), which provide both somatic and germ cells (Fig. 1C), cycle every 24-30 h, faster than the ESCs (Hobmayer et al., 2012; Buzgariu et al., 2014). As a result, animals transiently exposed to anti-proliferative drugs rapidly lose their ISCs and interstitial progenitors (collectively named i-cells) and progressively become nerve-free animals (Marcum and Campbell, 1978; Sugiyama and Fujisawa, 1978; Buzgariu et al., 2014). If force-fed, such epithelial *Hydra* remain viable and able to bud and regenerate likely due to the rapid adaptation of their ESCs, which modify their genetic program (Wenger et al., 2016).

By contrast, another *Hydra* species named *H. oligactis* (*Ho*) undergoes aging when the temperature drops to 10°C, a condition that induces gametogenesis, budding arrest and progressive animal degeneration (Brien, 1953; Littlefield et al., 1991; Yoshida et al., 2006). The massive production of gametes is accompanied by the loss of somatic interstitial cells, cytoskeleton disorganization, decline in body movements and feeding behavior (Yoshida et al., 2006; Tomczyk et al., 2015, 2019). Sexual animals from male and female *Ho* strains die within 4 months, showing Gompertzian mortality dynamics normally associated with aging (Finch, 1990) whereas *Ho* animals maintained at 18°C exhibit no signs of aging. Here, we used cold transfer to induce aging and analyze the cellular and molecular basis of aging in *Hydra*. We identified two *Ho* strains, one cold-sensitive (*Ho_CS*) that undergoes aging and another, cold-resistant (*Ho_CR*) that survives gametogenesis (Fig. 1D). In this study, we investigated the epithelial properties in these two strains and we found striking differences in epithelial stem cell renewal and autophagy efficiency in aging and non-aging animals.

## RESULTS

### The aging phenotype observed in *Ho_CS* in response to gametogenesis

To investigate aging in *Hydra*, we used two closely related male strains named *Ho_CR* and *Ho_CS*, which exhibit a similar budding rate at 18°C but respond differently to cold exposure (Fig.  1, Fig. S1). After cold transfer, ∼70% *Ho_CR* animals remained asexual and healthy and ∼30% reached sexual maturity within 25 days, then lost sexual traits and returned to physiological fitness without exhibiting aging signs (Fig. 1D,E, Fig. S1B). After 300 days at 10°C, all *Ho_CR* animals were healthy and budding, some showing mild dysmorphic signs, such as duplicated basal region and non-detached buds (not shown). By contrast, after transfer to 10°C, *Ho_CS* animals stopped budding within 4 weeks (Fig. S1B,C), differentiated testes for 90% of them and developed an irreversible aging phenotype that includes tentacle shrinking, head loss and stenosis of the body column as previously reported (Yoshida et al., 2006). Their survival time was negatively correlated to the testis number (Fig. S1D,E). Within 1 month, *Ho_CS* animals lost the ability to regenerate (Fig. 1F,G) and showed behavioral defects (Tomczyk et al., 2019) and aging became irreversible (Fig. S1F-N). Hence, *Ho_CS* but not *Ho_CR* animals undergo aging in response to cold-induced gametogenesis. We then decided to compare the properties of stem cells in these two strains, by selecting animals differentiating at least four testes.

### Impact of gametogenesis on somatic interstitial cells in *Ho_CS* and *Ho_CR*

**Fig. 2.**
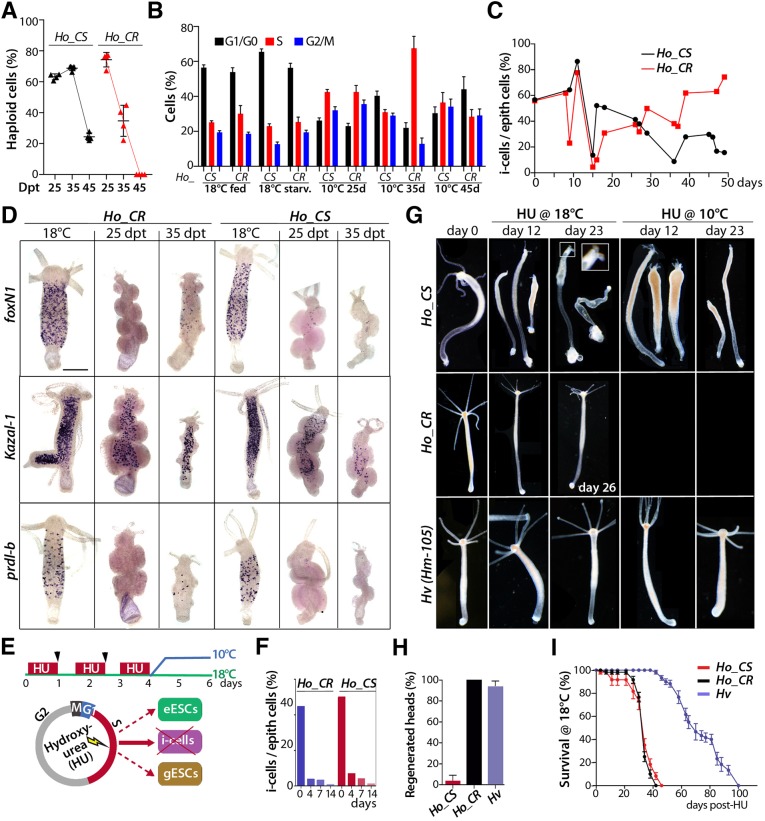
**Somatic interstitial loss upon gametogenesis-induced aging and pharmacological induction of aging in asexual *Ho_CS* animals.** (A,B) Modulations in haploid cell content (A) and cell cycle profiles (B) detected by flow cytometry in *Ho_CS* and *Ho_CR* animals maintained at 18°C or at 10°C for 25, 35 or 45 days; starv., 7-day starvation. Error bars represent s.d. (C) Fraction of interstitial cells (i-cells, single and pairs) over epithelial (epith) cells counted in macerated tissues (300 cells minimum per condition). (D) Expression of *foxN1* in i-cells, *Kazal-1* in gland cells and *prdl-b* in nematoblasts in *Ho_CS* and *Ho_CR* animals before and after transfer to 10°C (*n*=20 animals/condition). Scale bar: 300 µm. (E) Hydroxyurea (HU) treatment given as three successive 24 h pulses eliminates all cycling i-cells without affecting the ESCs, which cycle three to four times slower. Arrowheads indicate feeding times. (F) Fraction of i-cells over epithelial cells measured in *Ho_CR* and *Ho_CS* animals maintained at 18°C and analyzed before (day 0) or after HU treatment initiation (4, 7, 14 days). (G) HU-induced morphological changes noted in animals taken 12 or 23 days after day 0. Inset in the top row shows a magnification of the boxed area. (H) Head regeneration of *Ho_CS* and *Ho_CR* animals bisected 9 days after HU treatment initiation (*n*=3×10). (I) Survival rate of HU-treated *H_v_*, *Ho_CS* and *Ho_CR* animals (*n*=6×10). Error bars represent s.e.m. values in H,I. All HU treatments were performed as shown in E.

To monitor the impact of gametogenesis, we first analyzed the DNA content profiles of cells from *Ho_CS* and *Ho_CR* animals at various time points after transfer to 10°C (Buzgariu et al., 2014). At 25 days post-transfer (dpt) we noted a similar proportion of haploid and diploid cells and similar cell cycle profiles in both strains, at 35 dpt the number of haploid cells dropped significantly in *Ho_CR* but not in *Ho_CS*, and at 45 dpt haploid cells could no longer be detected in *Ho_CR* animals but were still present in *Ho_CS*, representing over 20% of all cells (Fig. 2A,B). At 35 dpt the fraction of S-phase cells was much higher in *Ho_CR* than in *Ho_CS* animals, suggesting a recovery of somatic interstitial proliferation. To confirm this transient versus sustained impact of gametogenesis on the stock of somatic i-cells, we quantified the proportion of i-cells and ESCs on macerated tissues over a 50-day period. In both strains we recorded a maximal decrease in the interstitial fraction at 15 dpt, followed by a complete recovery in *Ho_CR* by 40 dpt and a transient and partial recovery in *Ho_CS* (Fig. 2C)*.* These results indicate that gametogenesis leads to a massive loss of somatic interstitial cells in both strains, which is rapidly compensated for in *Ho_CR* but not in *Ho_CS*.

The expression patterns of *FoxN1*, *prdl-b* and *Kazal-1* in ISCs, nematoblasts and gland cells, respectively, confirmed the massive drop of expression at 25 dpt in both strains, followed by a recovery at 35 dpt in *Ho_CR* but not in *Ho_CS* (Fig. 2D). To monitor the recovery of somatic i-cells at the molecular level, we compared the RNA-seq profiles of 20 genes expressed in the interstitial lineages (Wenger et al., 2016). In *Ho_CS* and *Ho_CR* animals maintained at 10°C, we found 16/20 genes downregulated within the first 2 weeks after transfer, half of them showing a persisting low level of expression, and the other half showing at 35 dpt a secondary upregulation in *Ho_CR*, which was lacking or only partially observed in *Ho_CS* (*Cnnos2*, *cnox-2*, *foxN1*, *Notchl4*, *Pax-A*, *Cnvas1*, *Cnvas2*, *ZNF845*; Fig. S2A,B, Table S1).

We also compared the downregulation of these 20 interstitial-lineage markers in asexual *H. vulgaris* (*sf-1* strain) animals exposed to antiproliferative drugs [hydroxyurea (HU) and colchicine] or to heat-shock as reported by Wenger et al. (2016) with that measured in sexual *H. oligactis* strains: 11/20 genes exhibited a more drastic reduction of expression 10 days after drug or heat-shock exposure in *Hv_sf-1* than during gametogenesis in *Ho_CS* (see *CnASH*, *Cnnos1*, *Cnnos2*, *cnox-2*, *foxN1*, *myc1*, *NOWA*, *Pax-A*, *prdl-b*, *Hyzic*, *ZNF845* in Fig. S2C). This result indicates that the loss of somatic i-cells is more extensive after drug or heat-shock treatment in *Hv_sf-1* than in aging *Ho_CS* animals*.* Nevertheless, *Hv_sf-1* animals totally depleted of their i-cells remain fit whereas *Ho_CS* animals partially depleted of their i-cells undergo aging.

### Pharmacological induction of aging in *Ho_CS* in the absence of gametogenesis

Next, we tested the impact of interstitial cell loss in the absence of gametogenesis. We compared the respective responses to somatic i-cell loss in *Ho_CS* animals undergoing gametogenesis, and in asexual *Ho_CS* and *Ho_CR* animals maintained at 18°C and exposed to HU (Fig. 2E). We recorded a similar rapid global loss of i-cells in HU-treated *Ho_CS* and *Ho_CR* animals (Fig. 2F), which was actually faster than in animals maintained at 10°C (compare Fig. 2F with 2C). We also noted that *Ho_CS* but not *Ho_CR* animals exhibited an aging phenotype at 18°C when exposed to HU. This phenotype is similar to that observed after cold-induced gametogenesis although it was detected much earlier (Fig. 2G, upper row), indicating that gametogenesis is not necessary to promote an aging phenotypte in *Ho_CS.* By contrast, the *Ho_CR* animals exhibited a starvation phenotype after HU treatment, characterized by a lack of dysmorphic features and a reduced thickness and size, whereas *Hv* animals remained fit longer (Fig. 2G, middle and lower rows). *Hv* and *Ho_CR* animals were able to regenerate their head structures when bisected 9 days after HU treatment but *Ho_CS* were not (Fig. 2H). Despite these phenotypic differences, both *Ho_CS* and *Ho_CR* animals died within 6 weeks, whereas *Hv* animals were resistant to HU-induced i-cell depletion for twice as long (Fig. 2I). In *Ho_CR* the death occurred abruptly, whereas in *Ho_CR* it corresponded to a progressive deterioration.

To explain the low survival of *Ho_CR* animals after HU treatment at 18°C, we analyzed the bacterial load in each strain because in *H. oligactis* commensal bacteria populate the epithelial cells (Fraune and Bosch, 2007). We found the bacterial load in epithelial cells to be constitutively lower in *Ho_CR* than in *Ho_CS* but expanded upon HU treatment in *Ho_CR* epithelial cells (Fig. S3A). To test the deleterious effect of this bacterial load, we exposed HU-treated animals to an antibiotic cocktail (Wein et al., 2018) and recorded in *Ho_CR* cohorts an improved fitness and a prolonged survival (Fig. S3B,C), suggesting that the heavier bacterial load after HU impacts animal survival. In *Ho_CS* animals, the bacterial load remained low after HU and the antibiotic cocktail treatment was toxic.

### Epithelial response to interstitial cell loss in *Ho_CS*

**Fig. 3.**
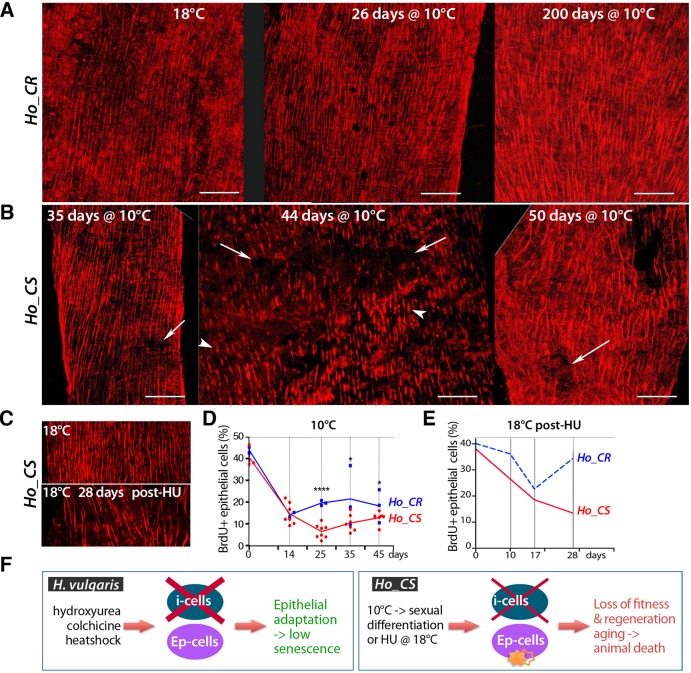
**Disorganization of the epithelial epidermal layer in aging *Ho_CS* animals.** (A,B) Phalloidin staining of the epidermis in *Ho_CR* and *Ho_CS* animals transferred to 10°C and fixed at the indicated time points. Arrows indicate disorganized regions of the epidermis, arrowheads indicate shortened myofibrils. Scale bars: 50 µm. (C) Phalloidin staining of the epidermis in control or HU-treated *Ho_CS* animals maintained at 18°C and fixed after 28 days. (D,E) BrdU-labeling index values measured after 96 h BrdU exposure performed at the indicated time points either after transfer to 10°C (D) or after HU treatment at 18°C as in Fig. 2E. In D, each dot corresponds to a replicate in which at least 300 cells were counted. In E, animals were maintained at 18°C. *P<0.05, ****P<0.0001 (unpaired *t*-test). (F) Scheme comparing the impact of i-cell loss in *Hv* animals, in which epithelial stem cells adapt (Wenger et al., 2016), and in *Ho_CS*, in which a more limited i-cell loss is lethal, suggesting a lack of epithelial adaptation.

Besides the i-cell loss, we also analyzed the global morphology of the epithelial layers in *Ho_CS* and *Ho_CR* animals, either after gametogenesis or after HU treatment (Fig. 3). In *Ho_CS* we detected a progressive disorganization of the muscle fibers, implying a loss of contractile function after 35 to 50 days at 10°C, whereas these myofibrils remained organized in *Ho_CR* (Fig. 3A,B). In *Ho_CS* animals exposed to HU at 18°C, we also recorded dramatic alterations of the myoepidermal network after 28 days (Fig. 3C). To test whether epithelial cell proliferation is modified during aging, we labeled live animals with bromo-2′-deoxyuridine (BrdU) for 96 h and quantified the proportion of BrdU-positive ESCs at various time points during cold exposure (Fig. 3D). From 40% BrdU^+^ cells at 18°C (day 0), we noted in both strains a rapid decline during the first 2 weeks post-transfer, followed by a stabilization with about 20% epithelial cells remaining cycling in *Ho_CR.* By contrast, in *Ho_CS* the stock of epithelial cycling cells dropped below 10% at day 25, and showed a slight recovery at later time points. Similarly, after HU treatment at 18°C, the stock of epithelial cycling cells first dropped during the first 2 weeks then became progressively re-established within 28 days in *Ho_CR* whereas it continued to decline in *Ho_CS* animals (Fig. 3E). At the molecular level, the upregulation of a large subset of cell cycle genes observed in *Ho_CR* animals was delayed in *Ho_CS* ones (Figs S4, S5, Table S2), which is in agreement with their inability to maintain epithelial proliferation after i-cell loss. In *H. vulgaris*, ESCs appear to adapt to i-cell loss (Wenger et al., 2016), therefore we hypothesized that the epithelial adaptation to i-cell loss is deficient in *Ho_CS*, a deficiency that contributes to the aging phenotype (Fig. 3F). As induction of autophagy is a key epithelial response to environmental changes in *Hydra* (Buzgariu et al., 2008; Chera et al., 2009), we decided to investigate the autophagy response in aging and non-aging *Hydra*.

### Starvation does not induce autophagy in *Ho_CS*

**Fig. 4.**
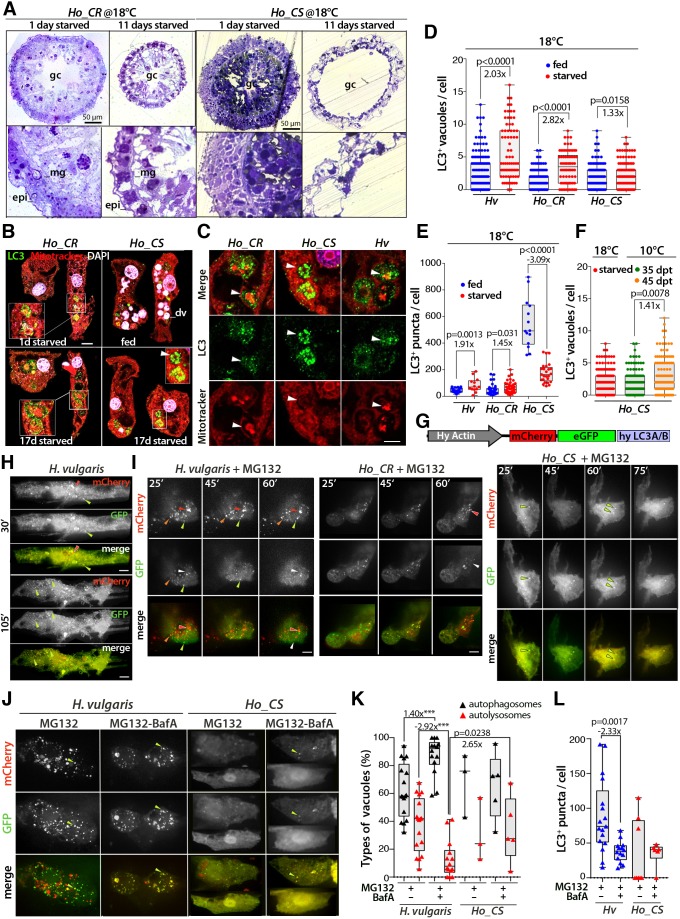
**Deficiency in the inducibility of the autophagy flux in ESCs from *Ho_CS* animals.** (A) Toluidine-stained transversal sections of gastric regions from 1- and 11-day starved animals. Lower panel show enlarged areas of the panels above. epi, epidermis; gc, gastric cavity; mg, mesoglea. (B) Detection of autophagic vacuoles (arrowhead) and digestive vacuoles (dv, pink) in epithelial cells immunostained for LC3 (green) and stained with MitoTracker (red) and DAPI (white). Scale bar: 10 µm. (C) Enlarged view of the LC3^+^ structures shown in B. Arrowheads indicate circular LC3^+^ structures surrounding sequestered portions of cytoplasm. Scale bar: 5 µm. (D,E) Number of LC3^+^ vacuoles (D) or LC3 puncta (E) in ESCs of regularly fed or 17-day starved *Hv*, *Ho_CR* and *Ho_CS* animals maintained at 18°C. (F) Number of LC3^+^ vacuoles in ESCs of *Ho_CS* maintained at 10°C for 35 or 45 days. (G) Structure of the mCherry-eGFP-hyLC3A/B dual autophagy sensor. (H,I) Live imaging of ESCs expressing the autophagy sensor in regularly fed *Hv*, *Ho_CR* or *Ho_CS* animals maintained at 18°C either untreated (H) or exposed to MG132 immediately before imaging (I). Green arrowheads indicate autophagosomes, orange arrowheads autophagosomes losing GFP fluorescence and red and white arrowheads autolysosomes. (J) Live imaging of ESCs expressing the autophagy sensor in *Hv* or *Ho_CS* animals exposed before imaging to MG132 for 3 h and to BafA (100 nM) for 16 h or not*.* Green arrowheads indicate autophagosomes. (K) Distribution of the LC3^+^ vacuoles between autophagosomes (black triangles) and autolysosomes (red triangles). (L) Number of LC3^+^ puncta in the cells shown in J. For box and whisker plots, the box indicates the 25th to 75th percentile, the line shows the median and the whiskers indicate the smallest and the largest value. Points represent individual values. *P*-values calculated using unpaired *t*-test. ***P=0.0003.

Autophagy is a dynamic flux that degrades intracellular components, a process essential for homeostasis and the stress response (Yu et al., 2018). Upon autophagy induction, double-membrane vesicles named autophagosomes form, which sequester part of the cytoplasm, then fuse with lysosomes to form autolysosomes that degrade their content. In *H. vulgaris* epithelial cells, the autophagy flux is enhanced upon starvation or stress (Buzgariu et al., 2008; Chera et al., 2009). As a first approach, we starved animals maintained at 18°C and noted a slower decrease in size and thickness in *Hv* animals, which lived twice as long as *Ho* ones (Fig. S6A,B). Despite the similar survival rate of starved *Ho_CS* and *Ho_CR* animals, we noted striking differences on histological sections, the former ones undergoing a dramatic reduction of their gastrodermis during an 11-day starvation (Fig. 4A). On sections analyzed by electronic microscopy, we could identify autophagosomes in *H. vulgaris* cells but rarely in *Ho_CS* ones (Fig. S6C). The ubiquitin-like protein ATG8/LC3, which is an essential autophagosome component (Birgisdottir et al., 2013), is encoded by two *LC3*-related genes in *Hydra* (*LC3A/B*, *LC3C*), predominantly expressed in ESCs (Figs S7 and S8).

To detect autophagosomes, we used an anti-human LC3B antibody that detects in one-day starved animals maintained at 18°C LC3-positive puncta and vacuoles (Fig. 4B,C). We defined puncta as small structures, approximately 1 μm in diameter, containing LC3 signal detected either in fixed cells after anti-LC3 immunolabeling or in live conditions in cells expressing the LC3-GFP-mCherry recombinant protein (Fig. 4G-L). By contrast, vacuoles are larger structures, several microns in diameter limited by a LC3^+^ rim that commonly surrounds a MitoTracker-positive core; they correspond to mature autophagosomes. Puncta were quantified by automated Imaris analysis whereas vacuoles were counted manually. After a 17-day starvation, the number of both vacuoles and LC3 puncta were significantly increased in *Hv* and *Ho_CR* animals but not in *Ho_CS* ones (Fig. 4D,E). Surprisingly, in daily-fed *Ho_CS* animals the number of LC3 puncta was constitutively high, decreasing with starvation, whereas in cold-maintained *Ho_CS* animals, which progressively stop eating, the number of LC3 vacuoles did not increase between day 0 and day 35, but increased moderately later between day 35 and day 45 (Fig. 4F). All these results suggest that autophagy regulation is altered in *Ho_CS*.

### Proteasome inhibition does not induce compensatory autophagy in *Ho_CS*

As in mammals, autophagy plays a crucial role in proteostasis and is upregulated upon proteasome inhibition (Kocaturk and Gozuacik, 2018), we used proteasome inhibition to induce autophagy in *Hydra*. We first tested the sensitivity of *Hydra* to the proteasome inhibitor MG132, and treated the animals either continuously over 6 days, or for a 16-h pulse (Fig. S9). In both conditions, *Ho_CS* animals showed signs of toxicity long before *Ho_CR* and *Hv* ones, suggesting a deficiency in compensatory autophagy. To visualize the autophagy flux, we modified the mCherry-eGFP-LC3 sensor produced by Pankiv et al. (2007). This chimeric protein is anchored in phagosomes, shows bi-fluorescence (green and red) in early and mature autophagosomes, but monofluorescence (red only) in autolysosomes, in which the pH-sensitive GFP fluorescence is quenched upon acidification after lysosome fusion (Pankiv et al., 2007). For the *Hydra* sensor, we coupled mCherry and eGFP to the *Hydra* LC3A/B protein under the control of the *Hydra* actin promoter (Fig. 4G). Animals electroporated with the mCherry-eGFP-hyLC3A/B plasmid expressed the chimeric protein for several days in a limited number of epidermal epithelial cells that were imaged 2 days after electroporation on whole intact animals in the presence of MG132.

In *Hv* and *Ho_CR* animals, the GFP fluorescence of most mCherry-GFP-hyLC3 puncta persisted in untreated animals (Fig. 4H) but vanished within 60 min of MG132 exposure, indicating an efficient activation of the autophagy flux (Fig. 4I). By contrast, in *Ho_CS* epithelial cells the fluorescence was constitutively diffuse and the rare mCherry-GFP-LC3 puncta remained stable in the presence of MG132. In epithelial cells exposed to MG132 in combination with bafilomycin A (BafA), which prevents lysosome fusion, the autophagic vacuoles remained stable in *Hv* and the diffuse LC3 pattern in *Ho_CS* remained unmodified (Fig. 4J). As expected, upon BafA treatment the proportion of autolysosomes decreased and the proportion of autophagosomes increased as they accumulated in the absence of autolysosome formation in *Hv* cells (Fig. 4K). The total number of LC3 puncta, including both the autophagosomes (green, yellow) and the autolysosomes (red), decreased in *Hv* cells after BafA treatment, suggesting that these cells form fewer autophagic vacuoles (Fig. 4L). In *Ho_CS* cells, the formation of LC3 puncta was limited although seemingly enhanced upon BafA treatment and the ratio of autophagosomes to autolysosomes did not vary (Fig. 4K,L). These *in vivo* assays confirm the poor inducibility of the autophagy flux in *Ho_CS.*

### Differential gene regulation of the autophagy machinery in *Ho_CS* and *Ho_CR*

In mammals, the autophagy machinery is tightly controlled at the transcriptional level by FoxO and TFEB (Füllgrabe et al., 2016). As this machinery is highly conserved in *Hydra* (Chera et al., 2009), we investigated gene regulation of the autophagy machinery in aging versus non-aging animals. Through a transcriptomic analysis performed in *Ho_CS* and *Ho_CR* animals, we compared the RNA-seq profiles of 75 genes linked to autophagy (Figs S10 and S11, Table S3). We found 51/75 genes specifically regulated at 10°C, 34/51 transiently upregulated in both strains but for 21/34 with a 10-day delay in *Ho_CS* compared with *Ho_CR*, 5/51 upregulated in *Ho_CR* but poorly in *Ho_CS* (*Ambra1*, *Atg16L1*, *Becn1*, *Rab24*, *Vamp7*), 4/51 transiently upregulated in *Ho_CS* but poorly in *Ho_CR* (*Atg2B*, *Atg4C*, *Plekhf2*, *Tollip*) and 7/51 upregulated at late time points in *Ho_CS* (*Atg4B*, *Atg7*, *Calr*, *DAPK1*, *LAMP1*, *NBR1*, *p62/SQSTM1*). These profiles indicate distinct regulations of the autophagy flux in *Ho_CS* compared with *Ho_CR*.

The lack of *Ambra1* and *Beclin-1* upregulation in *Ho_CS* points to a deficient initiation of phagosome formation at 10°C, whereas the stronger upregulation of *Ulk1/2* and *Atg13* in *Ho_CS* might reveal a possible regulatory feedback loop mechanism to activate the Ulk1 complex, which is crucial for activation of the Beclin-1 complex. In agreement with a deficient initiation, we noted a delayed upregulation of *Atg4B*, which encodes the protease required for LC3 cleavage, an essential step for autophagosome elongation. Also *Rab24*, which encodes a small GTPase required for terminating starvation-independent autophagy (Ylä-Anttila et al., 2015), shows a limited upregulation in *Ho_CS*. Finally, the late upregulation in *Ho_CS* of *NBR1* and *p62/SQSTM1* (named here *p62*) likely reflects a blockade of the autophagy flux. The shuttle receptor p62, which binds and brings polyubiquitylated proteins to proteasomal or autophagic degradation, accumulates when its cargo is not properly degraded (Seibenhener et al., 2004; Bjørkøy et al., 2005; Johansen and Lamark, 2011). In cnidarians, p62 is well conserved, is present as a single gene in *Hydra* and is predominantly expressed in ESCs (Figs S12 and S13A-C). Upregulation of *p62* in aging *Ho_CS* might be interpreted as an additional sign of inefficient autophagy flux.

### The p62/SQSTM1 protein is a marker of deficient autophagy in *Hydra*

**Fig. 5.**
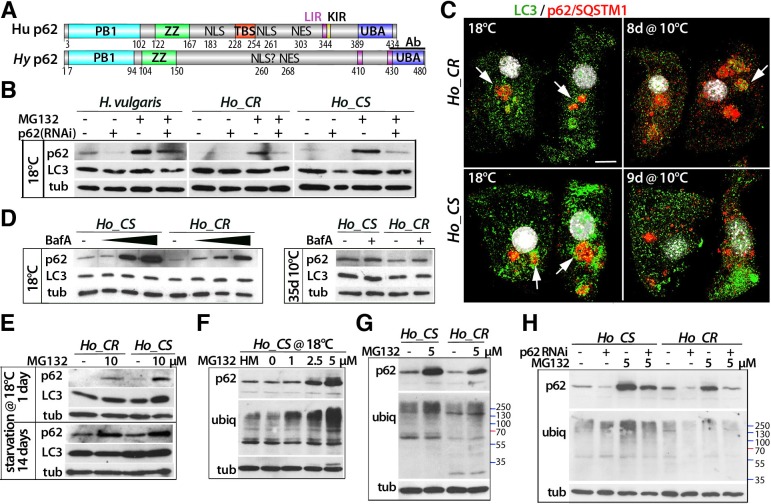
**Modulation of p62/SQSTM expression levels in *Ho_CS* animals.** (A) Structure of the human (Hu) and *Ho* p62/SQSTM1 (p62) proteins (see Fig. S12). ‘Ab’ indicates the region used to raise the anti-*Hydra* p62 antibody. (B) p62 levels in *p62*(RNAi) animals exposed or not to MG132; tub: α-tubulin. (C) Epithelial cells immunostained with the anti-*Hydra* p62/SQSTM1 (red) and the anti-human LC3 (green) antibodies, co-stained with DAPI (white). Arrows point to p62-labeled granules associated with LC3. Scale bar: 10 µm. (D,E) p62 levels in animals treated with increasing levels of BafA (D, left), or maintained at 10°C for 35 days and exposed or not to BafA (D, right), or starved for 1 or 14 days and exposed to MG132 (E). (F-H) p62 levels and ubiquitin patterns in animals exposed to MG132 (F,G), or knocked down for *p62* and exposed to MG132 (H). Except in D (right), all animals were maintained at 18°C and drug treatments given for 16 h.

To monitor the p62 regulation in aging animals further, we raised a polyclonal antibody against the *Hydra* p62 C-terminus (Fig. 5A, Fig. S12). As anticipated, p62 levels were lower in *p62*(RNAi) animals, and increased in MG132-treated animals (Fig. 5B). Also, p62 colocalized with LC3^+^ autophagosomes in immunocytological analyses (Fig. 5C, Movie 1). *Ho_CS* animals showed higher p62 levels than *Ho_CR* ones when maintained at 10°C (Fig. 5D), but also when starved at 18°C and/or treated with MG132 (Fig. 5E)*.* As expected, p62 accumulated when autophagy was inhibited with BafA, although to a much higher level in *Ho_CS* than in *Ho_CR* (Fig. 5D), indicating that autophagy contributes to p62 degradation in both *Ho_CS* and *Ho_CR* animals*.* As p62 binds polyubiquitylated (polyUb) proteins through its UBA domain, we analyzed whether an increase in p62 levels correlates with a parallel increase in polyUb proteins. At 18°C, MG132-treated *Ho_CS* animals exhibited a dose-dependent increase in both p62 and polyUb proteins (Fig. 5F, Fig. S14A); however, the polyUb pattern on western analysis was different between *Ho_CS* and *Ho_CR*, with high molecular bands more abundant in *Ho_CS* (Fig. 5G). Finally, we noted a decrease in the level of polyUb proteins in *p62*(RNAi) *Ho_CR* animals but not in *Ho_CS* ones, suggesting that the p62-dependent autophagy blockade induces an enhanced compensatory proteasome degradation in *Ho_CR* but not in *Ho_CS* animals (Fig. 5H). Hence, the accumulation of p62 protein in *Ho_CS* animals in response to physiological or pharmacological signals known to induce autophagy confirms the hypothesis of deficient autophagy flux*.* The parallel analysis of p62 and polyUb protein levels also confirms that the cross-talk between autophagy and proteasome degradation is less active in *Ho_CS* than in *Ho_CR*.

### Rapamycin delays aging and improves epithelial proliferation in *Ho_CS*

**Fig. 6.**
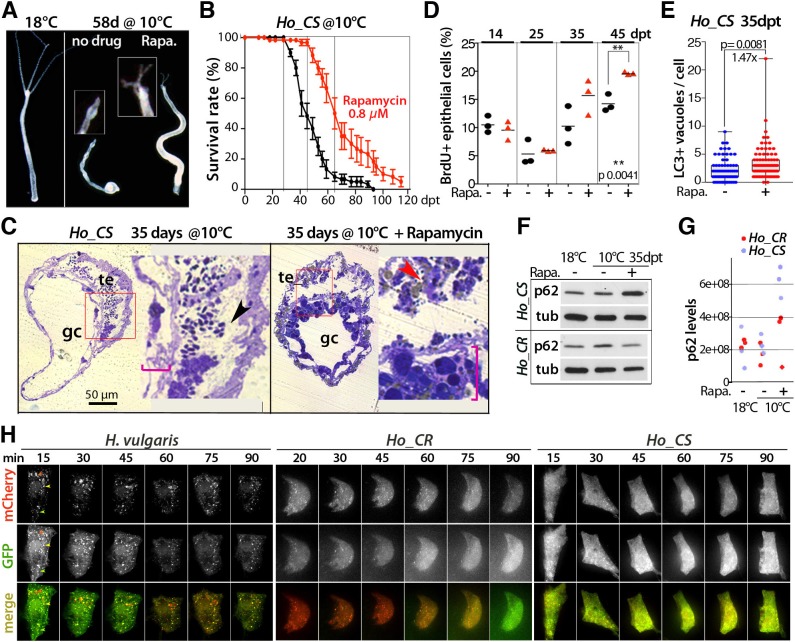
**Rapamycin treatment delays aging in *Ho_CS* without enhancing the autophagy flux.** (A,B) Aging phenotype at 58 dpt (A) and survival rate (B) in *Ho_CS* animals exposed, or not, to rapamycin from day 3 (dpt). Error bars represent s.e.m. (C) Toluidine-stained transversal sections of gastric regions from untreated (left) or rapamycin-treated (right) *Ho_CS* animals taken at 35 dpt. Red boxes indicate the enlarged areas shown on the right; pink brackets indicate gastrodermis thickness; black arrowhead indicates sperm cells in the testis lumen and red arrowhead indicates sperm cells engulfed in an epithelial cell. gc, gastric cavity; te, testis. (D) BrdU-labeling index values measured after 96 h BrdU exposure in *Ho_CS* animals maintained at 10°C in the presence (red triangles) or absence (black circles) of rapamycin. (E) Number of LC3^+^ vacuoles in ESCs of *Ho_CS* animals exposed (red dots) or not (blue dots) to rapamycin. Error bars represent s.d.; *P*-values calculated using the unpaired *t*-test. (F,G) p62 levels assessed by western blotting (F) or proteomic analysis (G) in *Ho_CR* and *Ho_CS* animals maintained at 18°C or transferred to 10°C and exposed to rapamycin for 35 days. tub: α-tubulin. (H) Live imaging of epithelial cells transiently expressing the mCherry-eGFP-hyLC3A/B autophagy sensor in animals maintained at 18°C and exposed to rapamycin (0.8 µM) from day 0. Arrowheads indicate newly formed autophagosomes with full GFP fluorescence and limited mCherry fluorescence (green); mature autophagosomes with double fluorescence GFP/mCherry (yellow); or autolysosomes with quenched GFP fluorescence (red).

In yeast, as in mammals, the macrolide compound rapamycin acts as a potent inhibitor of MTORC1 (Mechanistic Target of Rapamycin Complex 1), a complex that integrates signals coming from nutrients and energy levels. Through MTORC inhibition, rapamycin promotes resistance to starvation and induces autophagy, a necessary condition for lifespan extension (Bjedov et al., 2010; Fontana et al., 2010; Shen and Mizushima, 2014). To test the effect of rapamycin on *Hydra* aging, we continuously exposed *Ho_CS* animals to rapamycin from day 2 at 10°C and noted a significant improvement of the morphology, the contractility and the regenerative ability of treated animals (Fig. 6A, Fig. S14B). At 58 dpt, the head region, which was highly degenerated in untreated *Ho_CS* animals, remained equipped with tentacles in rapamycin-treated animals, which survived about 3 weeks longer (Fig. 6B). On histological sections, the attrition of the gastrodermis previously observed in aging animals (Fig. 3A) was reduced upon rapamycin treatment and the testes exhibited a flattened shape and contained fewer mature sperm cells (Fig. 6C, Fig. S14C). We measured the effect of rapamycin treatment on epithelial cell proliferation by monitoring the BrdU-labeling index at various time points after transfer to 10°C (Fig. 6D). The rapamycin treatment did not prevent the initial decline in ESC proliferation in *Ho_CS* but promoted the recovery of cell cycling as observed at 35 dpt and 45 dpt, when 20% ESCs were observed to be cycling, a level similar to that recorded in untreated *Ho_CR* animals (see Fig. 3D). We assume that the drastic reduction in ESC proliferation observed during gametogenesis or after HU treatment plays an important role in *Hydra* aging, and that rapamycin delays aging through the restoration of epithelial proliferation.

### Rapamycin does not enhance the autophagy flux in *Ho_CS*

Next, we tested whether the positive effects of rapamycin on health and lifespan in *Ho_CS* are mediated by a rescue of the deficient autophagic flux. In rapamycin-treated animals, we recorded at 35 dpt a modest increase (1.47×) in the number of LC3^+^ vacuoles (Fig. 6E), still p62 levels were increased in *Ho_CS* but lowered in *Ho_CR*, suggesting an efficient induction of autophagy in *Ho_CR* but not in *Ho_CS* (Fig. 6F). These results were confirmed by two independent quantitative proteomic analyses that showed an increase in p62 levels at 35 dpt in rapamycin-treated *Ho_CS* animals (Fig. 6G, Fig. S14D). *In vivo* analysis with the autophagy sensor showed a rapid activation upon rapamycin exposure of the autophagy flux in ESCs from *Hv* and *Ho_CR* but not in ESCs from *Ho_CS*, in which the mCherry-eGFP-LC3A/B fluorescence remained predominantly cytoplasmic with rare LC3^+^ puncta that persisted for 90 min after rapamycin exposure (Fig. 6H). All these results point to a persistent blockade of the autophagy flux in *Ho_CS* despite the presence of rapamycin, indicating that the positive effects of the rapamycin treatment on aging in *Ho_CS* are mediated via a distinct mechanism. We incidentally found that the epithelial cells from rapamycin-treated animals contain numerous sperm cells (Fig. S14E,F, Movie 2), pointing to a possible rapamycin-induced enhanced engulfment.

### The genetic blockade of autophagy in *H. vulgaris* impacts animal fitness and survival

**Fig. 7.**
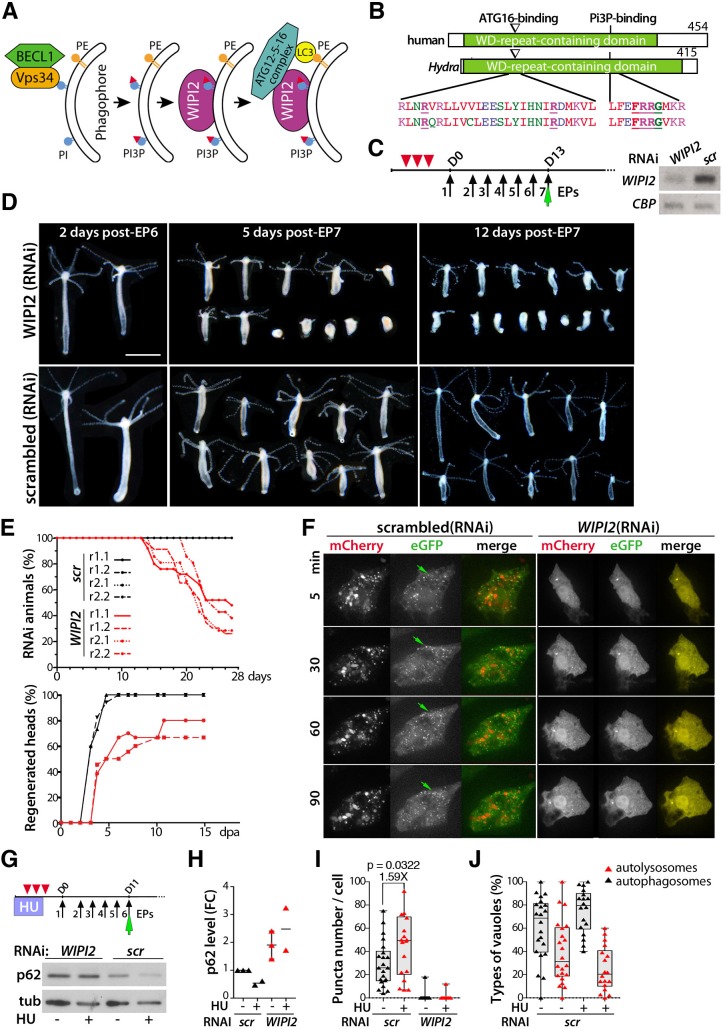
**Deficient autophagy flux upon *WIPI2* silencing in *H. vulgaris*.** (A) Scheme showing the role of WIPI2 in autophagosome formation (adapted from Dooley et al., 2014). (B) Structure of the human and *Hydra* WIPI2 proteins and alignment of the regions involved in ATG12-5-16 complex or PI3P binding. (C) Left: RNAi procedure used to knock down *WIPI2* expression in intact animals. Red arrowheads indicate feedings preceding siRNAi delivery, black arrows indicate siRNA electroporation (EP), green arrow indicates co-electroporation of the mCherry-eGFP-hyLC3 autophagy sensor on EP7*.* Right: *WIPI2* and *CBP* (CREB-binding protein) RNA levels measured by RT-PCR in animals exposed to *WIPI2* or scrambled (scr) siRNAs. D, day. (D) Phenotypic analysis of *WIPI2*(RNAi) and *scrambled*(RNAi) animals post-EP6 or -EP7. Scale bar: 1 mm. Note that all images have been cut and aligned on black background for ease of comparison. (E) Mortality rates recorded after EP7 and head regeneration efficiency in animals bisected at mid-gastric level 24 h after EP6. (F) Live detection of LC3 vacuoles in epithelial cells from *WIPI2*(RNAi) or *scr*(RNAi) animals 2 days post-EP7. MG132 added just before imaging. Green arrows indicate formation of GFP^+^ puncta in *scr*(RNAi) cells. (G-J) *WIPI2* silencing in HU-treated animals. (G) p62 and tubulin levels in *Hv* animals electroporated six times with *WIPI2*- or *scr*-siRNAs. (H) Fold change (FC) in p62 levels in animals exposed or not to HU and knocked down or not for *WIPI2*; each triangle represents a distinct experiment. (I,J) Imaris quantification of LC3 puncta (I), and autophagosomes and autolysosomes (J) in epithelial cells of *WIPI2*(RNAi) or *scr*(RNAi) animals expressing the autophagy sensor, imaged 2 days post-EP7. Vacuoles were quantified 3 h after MG132 exposure. For box and whisker plots, the box indicates the 25th to 75th percentile, the line shows the median and the whiskers indicate the smallest and the largest value. Points represent individual values. *P*-values calculated using the unpaired *t*-test.

As autophagy seems to play a pivotal role in the aging process of *Ho_CS*, we tested whether impairing autophagy in the non-aging *H. vulgaris* species promotes aging*.* To impair the autophagy process, we selected *WIPI2* for RNAi knockdown, a gene well conserved across metazoans (Fig. S15, Fig. S16A) that encodes the WD-repeat domain phosphoinositide-interacting protein 2, involved in the recruitment of the Atg12–5-16L1 complex, which is essential for LC3 conjugation and autophagosome formation (Dooley et al., 2014) (Fig. 7A). *Hydra* WIPI2 possesses both Atg16- and PI3P-binding motifs, which are necessary for its function (Fig. 7B, Fig. S15). To silence *WIPI2* that is ubiquitously expressed (Fig. S16B,C), we repeatedly electroporated *Hv_Basel* polyps with siRNAs and recorded an 80% reduction in *WIPI2* transcript level after 6 days of electroporation (EP6) (Fig. 7C). At the phenotypic level, we noted in *WIPI2*(RNAi) animals a decrease in size and an apical disorganization after EP7 (Fig. 7D) together with a high mortality rate recorded after 2 weeks (Fig. 7E). All control animals bisected after EP6 survived the amputation and regenerated their head within 5 days, whereas *WIPI2*(RNAi) animals exhibited a high mortality rate shortly after amputation (50-70%) and about 30% of survivors did not regenerate their head within 2 weeks (Fig. 7E).

The *in vivo* LC3 pattern in epithelial cells of *WIPI2*(RNAi) animals was diffuse and cytoplasmic, similar to that observed in *Ho_CS* epithelial cells (Fig. 7F). Also, the p62 level was twice as high (Fig. 7G,H) and the number of LC3 puncta dramatically decreased (Fig. 7I). These results indicate that a partial but sustained silencing of *WIPI2* suffices to block autophagy as evidenced by the switch from a dotted to a diffuse LC3 pattern. This autophagy blockade in *Hv* animals leads to a syndrome resembling the aging phenotype observed in *Ho_CS* animals. Finally, we tested whether the epithelial response to the loss of i-cells does require a fully functional epithelial autophagy in *H. vulgaris*. To do so, we exposed *Hv* animals first to HU and then to *WIPI2* siRNAs (Fig. 7G). In control RNAi animals, the HU treatment led to a decrease in p62 level (Fig. 7G,H), an increase in LC3 puncta (1.59×) together with a higher ratio of autophagosomes to autolysosomes (Fig. 7I,J), suggesting enhanced autophagosome formation. In *WIPI2*(RNAi) animals, HU treatment did not modify the higher p62 levels (Fig. 7G,H). These results indicate that the loss of i-cells tends to enhance epithelial autophagy in the non-aging *H. vulgaris*, but does not modify the autophagy partial blockade induced by *WIPI2* silencing.

## DISCUSSION

### The loss of interstitial stem cells triggers aging in the cold-sensitive strain of *H. oligactis*

**Fig. 8.**
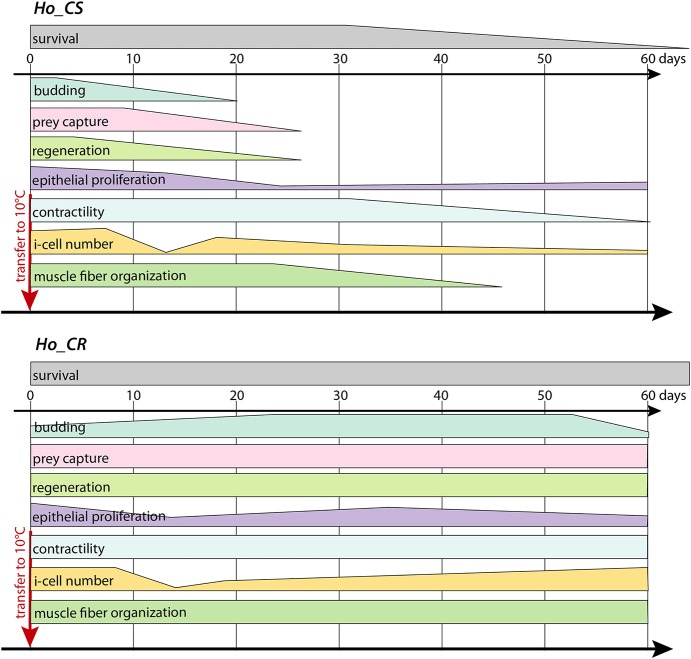
**Comparative view of the timing of the different signs of aging recorded in *Ho_CS* and *Ho_CR* sexual animals.** The data linked to survival are shown in Fig. 1E and Fig. S1, budding in Fig. S1, prey capture in Fig. 1C and Fig. S1N, regeneration in Fig. 1F,G, i-cell loss in Fig. 2C,D, epithelial cell proliferation in Fig. 3D, and Fig. S4A, animal contractility in Fig. S1M, and muscle fiber organization in Fig. 3A-C.

To investigate aging in *Hydra*, we used two *H. oligactis* (*Ho*) strains, one named *Ho_CS* (cold sensitive) that rapidly develops an aging phenotype when undergoing gametogenesis upon cold exposure as previously shown (Yoshida et al., 2006), and another that resists aging, therefore named *Ho_CR* (cold resistant). The developmental properties that characterize *Hydra*, i.e. budding and regeneration, were already irreversibly altered by 20 days after transfer to cold in *Ho_CS* animals but not in *Ho_CR* ones (Fig. 8). In both strains, the loss of somatic interstitial cells was massive after 2 weeks when spermatogenesis is maximal, and were progressively rescued in *Ho_CR* but not in *Ho_CS*. We assume that the massive gametogenesis prevents stocks of somatic i-cells from being replenished. In aging *Ho_CS* animals, the rapid loss of interstitial cell lineages led to the loss of *de novo* neurogenesis (Tomczyk et al., 2019), which is responsible for the loss of the feeding response, loss of contractility and progressive starvation. Similarly, epithelial proliferation was reduced within the first 2-3 weeks, a reduction that was spontaneously reversible in *Ho_CR* but not in *Ho_CS*. This aging phenotype shares features with mammalian aging, such as deterioration of the muscular network, neurodegeneration and global loss of health (Brien, 1953; Yoshida et al., 2006; Tomczyk et al., 2019; this work).

### Epithelial autophagy is required for maintaining epithelial self-renewal in *Hydra*

**Fig. 9.**
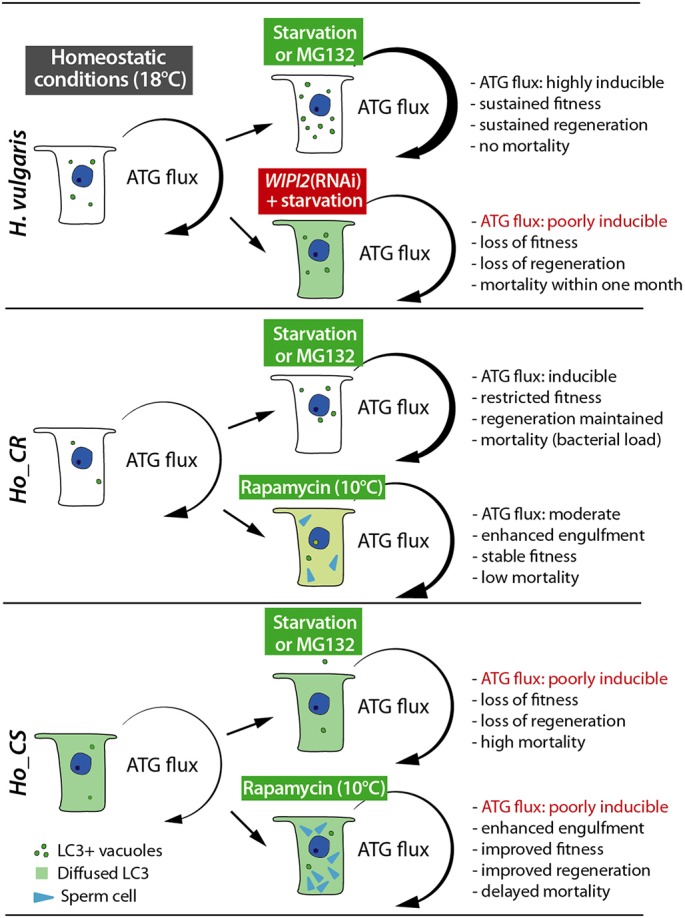
**The autophagic flux in epithelial cells of aging and non-aging *Hydra*.** Inducibility of the autophagy flux upon starvation, MG132 or rapamycin treatments as deduced from the formation of LC3-positive vacuoles and puncta *in vivo*, or the accumulation of p62/SQSTM1. Thickness of curved arrows indicate the relative extent of ATG inducibility.

A previous study had proposed that the massive loss of interstitial stem cells is actually the driving force for aging in *Ho_CS* (Yoshida et al., 2006). Here, we focused on the epithelial properties in *Ho_CS* versus *Ho_CR* and we found that the starvation induced by the loss of the feeding response in *Ho_CS* animals is not followed by a sustained autophagy in epithelial cells as observed in *Ho_CR* or *Hv* animals. This deficient autophagy in *Ho_CS* appears to be constitutive as observed in all contexts, either homeostatic or after induction upon starvation, proteasome inhibition by MG132, autolysosome formation inhibition by bafilomycin or inhibition of the TORC1 complex by rapamycin. These modulators provide convergent results that all support an inefficient induction of autophagy in *Ho_CS* (Fig. 9). This conclusion relies on investigations performed at multiple levels: (1) histological and cytological with the analysis of LC3^+^ vacuoles, (2) molecular with the fluctuations of p62/SQSTM1 levels and the regulations of the autophagy genes as static indicators of the autophagy flux, and (3) live imaging with the dual autophagy sensor, used here in an intact adult organism. We propose a scenario in which slow aging in *Hydra* requires an epithelial adaptation to environmental challenges through induction of an efficient autophagy that rescues epithelial cell cycling. Such a scenario is supported by: (1) the drop of epithelial self-renewal observed in animals that either undergo gametogenesis at 10°C or are exposed to HU at 18°C followed by an induction of the autophagy flux, which is efficient in *Ho_CR* but not in *Ho_CS* animals; (2) the rescue of epithelial cell cycling upon rapamycin-induced phagocytosis, which provides nutrients; and (3) the induction of aging in non-aging *Hv* animals upon blockade of autophagy through *WIPI2* silencing (Fig. 9). Therefore, in *Hydra* an inducible autophagy flux appears to be essential to maintain epithelial stem cell renewal.

### Compensatory autophagy induced by UPS blockade is deficient in *Ho_CS*

Autophagy has two main functions – to generate nutrients for the cell and to degrade altered intracellular proteins to maintain protein homeostasis (proteostasis), both functions being crucial for longevity (Cuervo, 2008; Morimoto and Cuervo, 2009). The maintenance of proteostasis relies on two main systems, the ubiquitin-proteasome system (UPS) and the lysosome-autophagosome, which are linked as autophagy can compensate the toxic effects of proteasome inhibition as shown in mammalian cells and *Drosophila* (Iwata et al., 2005; Ding et al., 2007; Pandey et al., 2007). To test the level of compensatory autophagy induced by UPS blockade in aging and non-aging *Hydra*, we used the proteasome inhibitor MG132 and we identified three distinct responses to UPS blockade (Fig. 9): in *Ho_CS* a rapid toxicity of MG132 and a lack of compensatory autophagy, with LC3 remaining mostly cytoplasmic; in *Ho_CR* a lower toxicity of MG132 and a moderate compensatory autophagy; in *H. vulgaris* a persisting fitness despite a prolonged exposure to MG132 and a high level of compensatory autophagy.

These responses reflect the expected correlation between the ability to enhance autophagy and the tolerance to proteasome inhibition and support the finding that autophagy is deficient in aging *Ho_CS*. In addition, the differences in MG132 tolerance between the *H. vulgaris* and *H. oligactis* species likely reflect a constitutive difference in proteostasis efficiency as previously reported (Gellner et al., 1992; Brennecke et al., 1998). The molecular chaperone Hsp70 is likely not similarly active in these two species, as *Hsp70* expression is upregulated in both species upon heat-shock but transcripts are unstable in *H. oligactis* and the Hsp70 protein not efficiently translated. At the evolutionary level, these results indicate that the link between autophagy and UPS is functional in non-bilaterian species. Given the multiple mechanisms proposed to integrate these two pathways (Korolchuk et al., 2010; Lamark and Johansen, 2010), *Hydra* provides a useful framework to trace their respective origins.

In conclusion, this study shows that the loss of somatic interstitial cells, linked or not to gametogenesis, provides a condition that reveals the efficiency in epithelial autophagy but is not the main cause of aging. In fact, the deficiency in epithelial autophagy, which remains to be characterized at the molecular level, appears to be the primary cause of aging, leading to reduced epithelial proliferation, loss of regeneration and, ultimately, animal death. This mechanism could be tested in the non-aging *H. vulgaris*, in which blocking autophagosome formation suffices to shorten animal lifespan. This mechanism shares similarities with the role of autophagy in aging mammals, in which autophagy is crucial for the survival of stem cells (Boya et al., 2018), including hematopoietic stem cells (Warr et al., 2013; Gomez-Puerto et al., 2016) and muscle satellite cells (García-Prat et al., 2016). These findings point to autophagy as an evolutionarily conserved robust anti-aging mechanism that contributes to the preservation of adult stem cells.

## MATERIALS AND METHODS

### *Hydra* strains, *Hydra* culture, monitoring of aging and behavioral analyses

*Hydra vulgaris* from the Basel or *sf-1* strains (*Hv_Basel*, *Hv_sf-1*) and two *Hydra oligactis* (*Ho*) strains, cold-sensitive (*Ho_CS*), cold-resistant (*Ho_CR*), were mass cultured at 18°C±0.5°C in Hydra Medium (HM; 1 mM NaCl, 1 mM CaCl_2_, 0.1 mM KCl, 0.1 mM MgSO_4_, 1 mM Tris-HCl pH 7.6). Animals were fed three to four times a week with freshly hatched brine shrimps (*Artemia*; Watson Aquaculture). To induce gametogenesis, *Ho_CS* and *Ho_CR* animals were transferred to 10°C±0.3°C and then fed twice a week. To monitor aging six sets of ten animals per condition placed in six-well plates (Greiner Bio-One) were observed five to seven times a week at the stereomicroscope and imaged at least once a week. Budding animals were counted daily and detached buds were removed. The reversibility of aging was tested on cohorts of 20 animals. For touch responsiveness, animals were stimulated with tweezers in the peduncle region and observed under the binocular microscope. For testing prey ingestion, animals were observed at the stereomicroscope 1 h after feeding and counted as positive when at least one *Artemia* was found in the gastric cavity.

### Drug treatments and regeneration experiments

To deplete cycling cells at 18°C, cohorts of 20 animals were exposed to N-hydroxyurea (HU, Applichem, 5 mM diluted in HM) as indicated in Fig. 2E. The post-HU antibiotic treatment was given as an antibiotic mix containing ampicillin, rifampicin, streptomycin and neomycin (50 mg/ml each) as reported previously (Wein et al., 2018). This treatment was given continuously from day 2 after HU release and changed every other day. Untreated animals were kept in HM containing 0.01% DMSO during the same period. For rapamycin treatment, animals were continuously exposed to rapamycin (LC Laboratories, 0.8 µM diluted in HM) from 3 dpt to 10°C, the drug being changed three times a week. To inhibit proteasomal degradation, animals were treated with the proteasome inhibitor MG132 (SelleckChem, 5 µM diluted in HM, DMSO 0.05%) either for 16 h, or over a 5-day period at the indicated concentrations. To test the effect of these drugs on head regeneration, two sets of 15 animals per condition fed twice a week prior to bisection were starved for 2 days, bisected at the mid-gastric position and monitored over 13-15 days. For bafilomycin A (Enzo Life Sciences) treatment, animals were exposed to 50, 100 or 200 nM solution for 16 h diluted in HM, DMSO 0.01%.

### mRNA *in situ* hybridization and phalloidin staining on whole mounts

For mRNA whole-mount *in situ* hybridization (WM-ISH) 2-day starved animals were relaxed for 60 s in 2% urethane, fixed overnight at 4°C in 4%paraformaldehyde (PFA) prepared in HM pH 7.5 (PFA-HM) and stored in methanol at −20°C. Transcripts were then detected with specific riboprobes as described by Bode et al. (2008). To visualize actin fibers, animals were starved for at least 24 h, washed in HM, relaxed for 60 s in 2% urethane, fixed for 4 h in PFA-HM, permeabilized in 0.1% Triton X-100 in PBS (PBS-TX) for 15 min, stained for 1 h in phalloidin-Alexa Fluor 546 1:100 (Invitrogen), washed in PBS, DAPI-stained (0.2 µg/ml) for 30 min, washed in PBS, washed briefly in water, mounted in Mowiol and imaged using Zeiss LSM700 or Leica SP8 confocal microscopes.

### Histological sections and transmission electronic microscopy (TEM)

Animals were fixed in Fixative-1 [2% glutaraldehyde, 4% formaldehyde, 0.1 M 2AsO_2_Na (pH 7.4), 2 mM CaCl_2_, 150 mM sucrose] for 30 min at room temperature (RT), then overnight at 4°C, then washed three times in pre-cooled 0.1 M sodium cacodylate, transferred to Fixative-2 [1% OsO_4_, 0.8% K_3_Fe(CN)_6_, 0.1 M 2AsO_2_Na (pH 7.4)] for 60 min on ice, then washed three times in 0.1 M 2AsO_2_Na, five times in H_2_O and stained in 1% uranyl acetate for 90 min in the dark. After stepwise ethanol dehydration, samples were washed three times in 100% C_3_H_6_O, incubated overnight in 1:1 propylene oxide Epon resin solution (25.7 ml Agar 100 resin, 9.3 ml dodecenylsuccinic anhydride, 16.5 ml NMA, 1.6 ml BDMA from Agar Scientific), then three times for 120 min each in fresh 100% Epon embedding medium, transferred into embedding molds and cured for 48 h at 60°C. Resin blocks were cut with a Leica UCT ultramicrotome, 70 nm ultrathin sections were transferred onto 100-mesh copper grids, stained for 5 min in 2% uranyl acetate (in 1:1 water-acetone), for 10 min in lead citrate (25 mg/ml in H_2_O), observed with a Tecnai G2 transmission electron microscope at 120 kV accelerating voltage and imaged with a Tietz TemCam F214 CCD camera. For histological analysis, sections were dried on a glass slide, covered with a puddle of Toluidine Blue solution (1% Toluidine Blue, 1% sodium borate in water) and heated on a hot plate (70°C) for 30-120 s, or until the puddle steams. Excess stain was gently washed off with distilled water, and slides were dried and mounted in Mowiol.

### *Hydra* p62/SQSTM1 antibody production and immunodetection on macerated tissues

A mouse polyclonal antibody was raised against *Hydra* p62/SQSTM1 by Delphi Genetics (Charleroi, Belgium) against the C-terminal stretch KESKLERSLRQMEAMGFDNEGGWLRQLLISKDCSIDKVLDALSPAK (for validation of this antibody see Fig. 5B and Fig. S13D). The corresponding DNA sequence was synthesized following the mammalian codon usage, then inserted into the pStabyBoost expression vector and electroporated *in vivo* into mouse muscle. Sera were collected from five mice after three immunizations. For immunodetection, three to seven animals per condition were macerated as described by David (1973), cells were then spread on Superfrost Plus slides (Thermo Scientific), dried for 2 days at RT, washed in PBS, blocked in BSA-TX (bovine serum albumin 2%, Triton X-100 0.1%) for 2 h, incubated overnight at 4°C with the following antibodies: anti-ubiquitin (1:200, FK2 antibody, BML-PW0755-0025, Enzo Life Sciences), anti-LC3B (1:300, Novus Biologicals, nb100-2220, raised against the 1-100 N-terminus human LC3B), anti-*Hydra* p62/SQSTM1 (1:200), anti-tubulin (1:300, T5168, Sigma-Aldrich) (see Table S6). Slides were then washed in PBST [PBS (pH 7.2), Tween-20 0.1%] incubated in anti-rabbit Alexa Fluor 488 and/or anti-mouse Alexa Fluor 555 antibodies (1:600, Invitrogen, A-21206 and A31570), counterstained with DAPI and MitoFluor Red589 (1:5000, Life Technologies) and imaged as above. Autophagic vacuoles were counted in 100-300 cells per condition.

### BrdU labeling and immunodetection on macerated tissues

30 polyps per condition were exposed to BrdU (5 mM in HM, Sigma-Aldrich) for 96 h or as indicated. After BrdU exposure, animals were washed in HM and the body columns of three sets of three animals per condition were dissected, macerated as independent triplicates, immunodetected with the BrdU Labeling and Detection Kit-I (Roche) and DAPI counterstained. Samples were imaged using a Leica D5550 fluorescence microscope, and at least 300 epithelial cells were counted on each replicate to calculate the BrdU-labeling index. The same slides were used to count the interstitial to epithelial stem cell ratio (ISCs/ESCs). For sexual animals, only those bearing at least four testes were selected.

### *In vivo* detection of the autophagy flux with the mCherry-GFP-LC3A/B autophagy sensor

The mCherry and eGFP sequences were codon optimized for expression in *Hydra* (GenScript) and inserted into the hoTG vector after removal of the original GFP sequence (Wittlieb et al., 2006). The hoTG vector contains 1386 bp of the *Hydra* Actin promoter and a 677 bp-long terminator from the *Hydra* Actin gene. The *Hydra* LC3A/B sequence was amplified from *Ho_CS* cDNA and inserted into the pGEM-T vector. Coding sequences of mCherry, eGFP and hyLC3A/B were amplified from the hoTG_mCherry, hoTG_eGFP and PGEM-T_hyLC3A/B plasmids, respectively, and inserted in-frame into the hoTG vector following the Gibson method (Gibson et al., 2009). See Table S4 for primer sequences. To monitor the autophagy flux, 20 regularly fed animals were electroporated 8 h after feeding with a GenePulserXcell (Bio-Rad) delivering two pulses of 150 V at 1-s intervals, 50 ms each as described by Watanabe et al. (2014). Animals were immediately transferred from the electroporation cuvettes to six-well dishes in HM for recovery. Forty hours later, animals displaying fluorescent cells in the gastric region were relaxed in dishes containing 1% urethane HM. Unless otherwise specified, animals were incubated in MG132 (5 µM) for 3 h before live imaging was performed with a 3i spinning disc confocal microscope with laser power 75, exposure time 100 ms for *Hv* and *Ho_CR* animals; laser power 50, exposure time 50 ms for *Ho_CS* animals; camera intensification 250 in all conditions.

### Whole cell extracts (WCE) and western analyses

WCE were prepared from 20-100 polyps starved for 2 days, washed in HM, resuspended in lysis buffer [SDS 2%, Tris-HCl pH 8.0 100 mM containing a mix of protease (Roche complete cocktail) and phosphatase inhibitors (Biotools cocktail or lab-made cocktail: 8 mM NaF, 20 mM β-glycerophosphate, 10 mM Na_3_VO_4_, 0.1 mg/ml PMSF)] and passed through a 0.6×30 mm syringe needle, yielding 100-500 µg proteins per sample. For each condition, 10-30 µg WCE were loaded on a 12% Rotiphorese Gel 30 (Roth), transferred to Hybond-P membrane (GE Healthcare) and detected with the antibodies raised against LC3B (1:1000, Novus Biologicals, nb100-2220), *Hydra* p62/SQSTM1 (1:1000, lab-made), ubiquitin (1:2000, Abcam, ab137025) and α-tubulin (1:2000, Sigma-Aldrich, T5293).

### RNA interference (RNAi) procedure

RNAi was performed by electroporating siRNAs as previously reported (Watanabe et al., 2014) with minor modifications. Briefly, 30 animals were electoporated as above either with a ‘scrambled’ siRNA (4 µM, diluted in water) used as control, or with a mixture of three siRNAs (Eurofins Genomics) targeted against *p62/SQSTM1* or *WIPI2* (final concentration 4 µM in water, see Table S5 for sequences). The procedure was repeated every other day as indicated.

### Transcriptomic analyses

RNAs were extracted from pools of 20 *Hydra* using the RNeasy kit (Qiagen) (see Fig. S2A for conditions). Libraries were prepared with the Low Sample TruSeq total RNA preparation protocol using 15 PCR cycles. Library concentrations were measured with a Q-bit (Life Technologies). Pools of seven multiplexed libraries were loaded on a HiSeq 2000 sequencer (Illumina) and single-end sequenced using the standard 100 bp protocol. On average, 28 mio reads (3 mio s.d.) were obtained and cleaned for remaining adapters using cutadapt v1.9.1 (Martin, 2011). Assemblies were done separately for *Ho_CR* and *Ho_CS*, using Trinity v2.2.0 (Grabherr et al., 2011) with the *in silico* normalization option. These contigs were translated into protein sequence, keeping the longest open reading frame (ORF) per contig and excluding all proteins with ORFs <100 residues. Translated sequences were subjected to one round of clustering using Cd-hit (Li and Godzik, 2006) with options –c1 and –s1, which remove strictly identical protein coding sequences. After this step, for *Ho_CR* there remained 44,490 sequences and 24,590 Trinity ‘genes’, and for *Ho_CS* 45,002 sequences and 23,128 Trinity ‘genes’. Reads were cleaned with cutadapt (see above) and abundances were estimated by using Salmon (Patro and Kingsford, 2015). Quantification outputs were extracted, summarized and normalized using tximport and DESeq2 as described by Soneson et al. (2016). These normalized counts per gene were used to produce heat maps and plots. For heat map representations, each value corresponds to the median number of reads obtained at given 10°C time points divided by the number of reads measured at the corresponding time point at 18°C, except for the day 35 10°C time point, which was normalized using the day 32 18°C value. After log2 transformation, data were standardized by strain using time points from day 7 to day 35. Orthologies between *Ho_CR* and *Ho_CS* transcripts/genes were assigned manually. Sequences and RNA-seq profiles are available at https://hydratlas.unige.ch/blast/datasets/normacounts_CR2017.csv.gz and https://hydratlas.unige.ch/blast/datasets/normacounts_CS2017.csv.gz.

### Proteomic analyses

The dimethyl stable isotope labeling protocol (Boersema et al., 2008, 2009) was used to quantify relative protein levels in *Ho_CS* polyps maintained either at 18°C (control), or at 10°C for 35 days (aging), or at 10°C for aging but exposed to rapamycin (rapamycin aging). For each condition, 10 µg WCE were digested by filter-aided sample preparation (FASP) (Wiśniewski et al., 2009) with minor modifications. Dithiothreitol (DTT) was replaced by Tris(2-carboxyethyl)phosphine (TCEP) as reducing agent and iodoacetamide by chloroacetamide as alkylating agent. Proteolytic digestion was first performed with Endoproteinase Lys-C followed by a second trypsin digestion. Next, peptide samples were labeled on StageTips with isotopomeric tags as follows: control samples were labeled with light dimethyl reactants (CH_2_O+NaBH_3_CN), aging samples were labeled with medium dimethyl reactants (CD_2_O+NaBH_3_CN) and rapamycin aging samples were labeled with heavy dimethyl reactants (13CD_2_O+NaBD_3_CN). Independently labeled peptide samples were subsequently mixed in equal (1:1:1) ratio and fractioned into six fractions by strong cation exchange (SCX) chromatography on StageTips (Kulak et al., 2014). For LC-MS/MS measurements, each SCX fraction was again separated by C18 Reverse Phase nano UPLC using a Dionex Ultimate 3000 RSLC system (Thermo Fisher Scientific) on line connected to an Orbitrap Elite Mass Spectrometer (Thermo Fisher Scientific). Samples were first trapped on a home-made capillary pre-column (Magic AQ C18; 3 µm-200 Å; 2 cm×100 µm ID), then separated on a C18 capillary column (Nikkyo Technos Co.; Magic AQ C18; 3 µm-100 Å; 15 cm×75 µm ID) using a gradient ranging from 99% of solvent A (2% acetonitrile and 0.1% formic acid) to 90% of solvent B (90% acetonitrile and 0.1% formic acid). The Source spray voltage was set at 1.6 kV, and capillary temperature at 250°C. Acquisitions were performed using a Data-Dependent mode; the 20 most intense parent ions were selected for subsequent fragmentation by collision induced dissociation (CID) into the Linear Ion Trap and then excluded for the following 40 s. Orbitrap MS survey scan (*m/z* 300–1800) resolution was set to 120,000 (at 400 m/z) and fragments were recorded with low-resolution Rapid mode on the Trap. Filling times were set as follows: MS mode, 200 ms with a limit of 1×10^6^ ions and MS^2^ mode, 50 ms with a limit of 5×10^3^ ions. Only multiply charged ions were allowed for fragmentation when reaching a threshold of 1000 counts, and during fragmentation an activation *Q* value of 0.25 was used and the normalized collision energy was set at 35% for 10 ms. For protein identification, quantification and bioinformatics, data were processed using MaxQuant software (Cox and Mann, 2008) (v1.5.1.2) as described by Chopra et al. (2014). MS/MS spectra were searched against the conceptually translated *de novo* transcriptome protein database described above. Statistical processing and outputs displays were performed using the embedded MaxQuant tool Perseus (Cox and Mann, 2012) and home-made programs written in R.

### Statistical analyses

There were no statistical or computational methods involved in the determination of the sample size. The sample size, when applicable, is indicated in the figure legend or Materials and Methods section. Biological replicates come from fully independent experiments performed on different days. Technical replicates are a series of samples of a given experiment, all samples collected the same day (usually three or four). The number of replicates is indicated in the Materials and Methods section. As a general rule, we tried to perform at least three independent experiments. We did not exclude any data from the analyses. When applicable, the statistical methods we used are described in the Materials and Methods section or in the figure legends. For most results, raw data from the replicates are plotted directly in the figure. For the plots shown in Figs 2A,B, 4D-F,K,L, 6E and 7I,J, average values with s.d. are shown; for the plots shown in Figs 2H,I and 6B, average values with s.e.m. values are represented. The raw data for Fig. 2I and Fig. 6B are shown in Table S7 and Table S8, respectively.

## Supplementary Material

Supplementary information
