# Supplementary material for: Deficient autophagy in epithelial stem cells drives aging in the freshwater cnidarian Hydra
Source: Development. 2020 Jan 23;147(2):dev177840. doi: 10.1242/dev.177840 (PMC6983715; doi:10.1242/dev.177840)
Supplement: Supplementary information [file develop-147-177840-s1.pdf]

SUPPLEMENTARY FIGURES

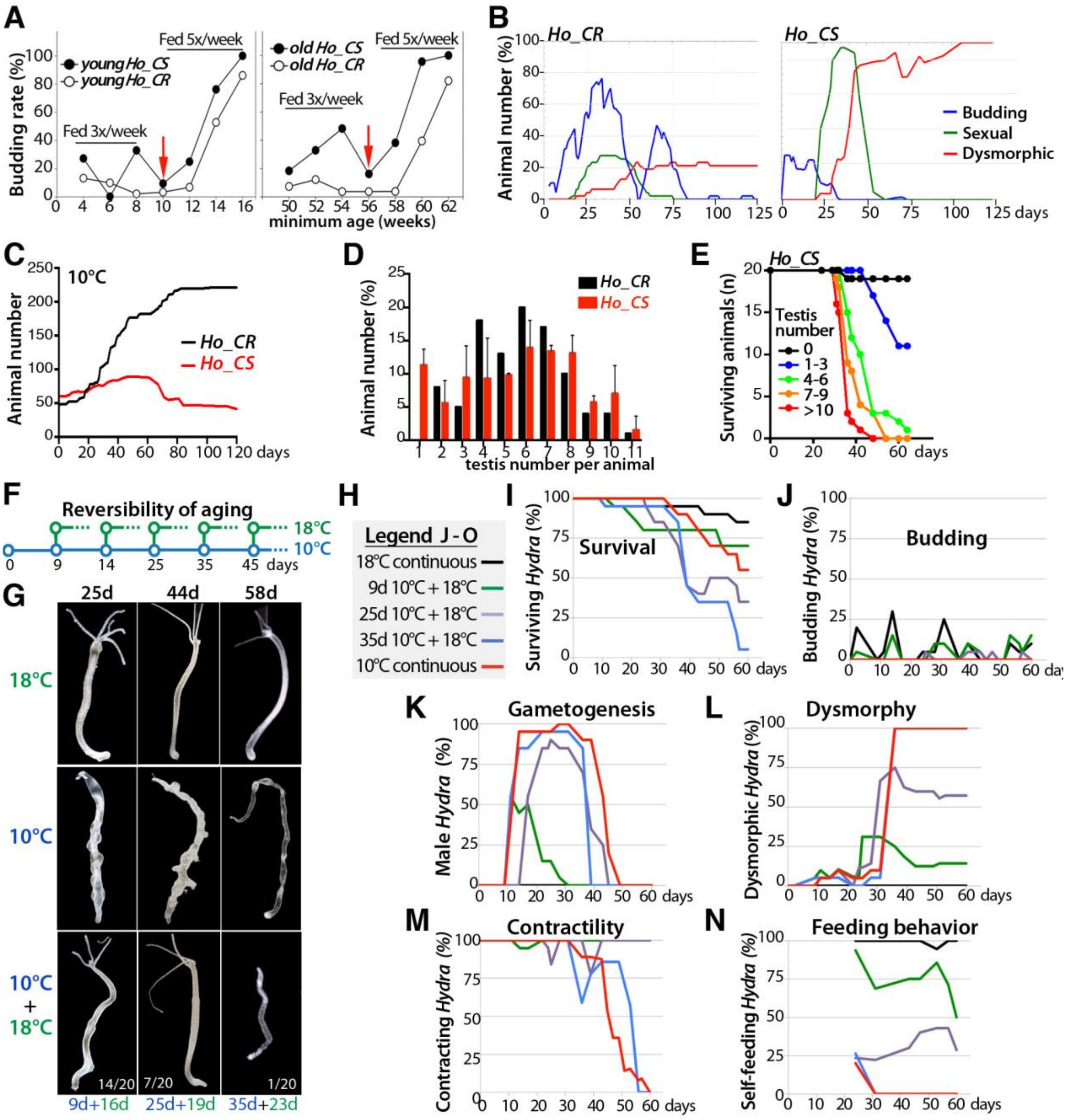

Figure S1: Features and reversibility of the cold-induced aging phenotype in *Ho_CS* animals

(A) Budding rate of juvenile (4 to 16 weeks old, left graph) and older (50 to 62 weeks old, right graph) *Ho_CS* and *Ho_CR* animals kept at 18°C and submitted to two successive feeding regimes. Red arrows indicate the transition from 3x to 5x feedings a week. In both cohorts the budding rate is up-regulated by a heavy diet, however older *Ho_CS* animals appear more prone to bud than *Ho_CR*. **(B,C)** Comparative analysis of two cohorts of *Ho_CR* (n=48) and *Ho_CS* (n=60) animals transferred to 10°C on day-0, showing in (**B**) the rates of budding (blue), sexual differentiatIon (green) and dysmorphic traits (red), and in (**C**) the population size kinetics. Buds produced at 10°C do not undergo aging. In the experiment depicted in (**C**), buds were not removed from the culture and thus included in the population size. The recorded dysmorphic features were duplicated head or foot regions, and arrested budding process in *Ho_CR*, tentacle shrinking, head loss, body column stenosis in *Ho_CS*. **(D)** Similar distribution of testis number in *Ho_CS* and *Ho_CR* cohorts maintained at 10°C for 25 days. Animals that did not develop testes were not included. **(E)** Survival of *Ho_CS* animals according to the number of testes they produce. **(F)** Scheme showing the procedure for testing the reversibility of aging. At day-0 seven *Ho_CS* cohorts (for each cohort n=20)

Development • Supplementary information

were separated from the 18°C main culture, one was maintained at 18°C (top line) whereas the others were transferred to 10°C. At each indicated time-point, one cohort was moved back to 18°C, while one cohort remained at 10°C throughout the experiment (blue bottom line). Animals were fed twice a week all through the experiment.

(G) Representative phenotypes of animals maintained either at 18°C (upper row) or at 10°C (middle row) or moved from 10°C to 18°C at day-9, day-25 or day-35 (lower row). The fraction of animals appearing healthy when returned from 10°C to 18°C is 70% (14/20), 35% (7/20) after 5% (1/20) respectively. After 35 days at 10°C, animals no longer recover, the single animal still alive 23 days after the switch back to 18°C died in the following days. Approximately 50% animals returned to 18°C at day-9 had shown first signs of sexual traits. Upon return to 18°C, testes of these animals stopped develop and resorbed. **(H-N)** Observed percentages of surviving (I), budding (J), sexually differentiating (K), dysmorphic (L), or touch-responsive (M) animals when maintained at 10°C over 60 days. All parameters were recorded five times a week except the feeding behavior (N) recorded only twice. For measuring the survival rate, buds produced during that period were removed from the culture soon after detachment, thus not included in the total animal number (I). The observed peaks of budding are caused by the feeding rhythm (twice a week, J). Contractibility was measured by stimulating briefly the peduncle region with tweezers and the percentage of animals contracting upon stimuli was recorded (M). The efficiency of the feeding behavior was assessed one hour after feeding as the percentage of animals with preys inside the gastric cavity (N). Animals able to catch preys with tentacles but unable to transfer it to the gastric cavity were excluded.

Development • Supplementary information

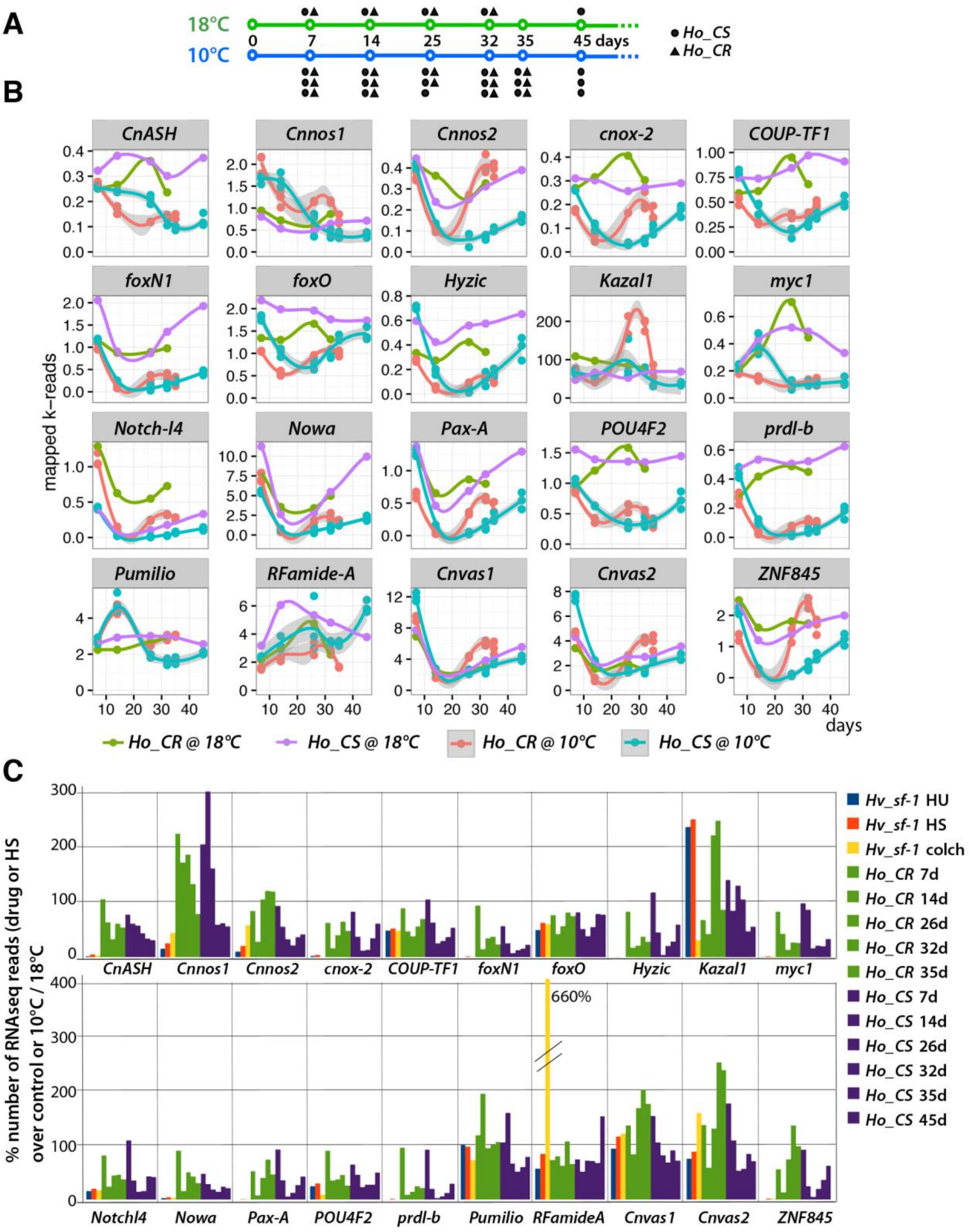

Development • Supplementary information

Figure S2: RNA-seq profiles of 20 genes expressed in interstitial cell lineages in *Ho_CS* and *Ho_CR* animals maintained at 18°C or transferred to 10°C.

(A) Scheme describing the procedure used for quantitative RNA-seq analysis of aging. RNA samples were collected at indicated time points from *Ho_CS* and *Ho_CR* animals either maintained as a unique cohort at 18°C or as three distinct parallel cohorts at 10°C. **(B)** Individual RNA-seq profiles of 20 evolutionarily-conserved genes predominantly expressed in the interstitial cell lineage in *H. vulgaris* as described in ref. (Wenger et al., 2016). See the access of the corresponding sequences in **Table-S1**. Note the drastic but transient down-regulation of most genes in *Ho_CR* and *Ho_CS* animals maintained at 10°C, highlighting the partial elimination followed by the recovery of the corresponding cell types. **(C)** Comparative RNA-seq analysis of i-cell gene expression in *Hv_sf-1* animals 10 days after exposure to HU (blue), heatshock (HS, red), colchicine (yellow), or in *Ho_CR* (green) and *Ho_CS* (purple) at various time points after transfer to 10°C. Values were normalized on values measured in untreated *Hv_sf-1* animals (blue, red, yellow and green values) or in *Ho* animals maintained at 18°C (purple). All data are available on HydrATLAS.unige.ch.

Development • Supplementary information

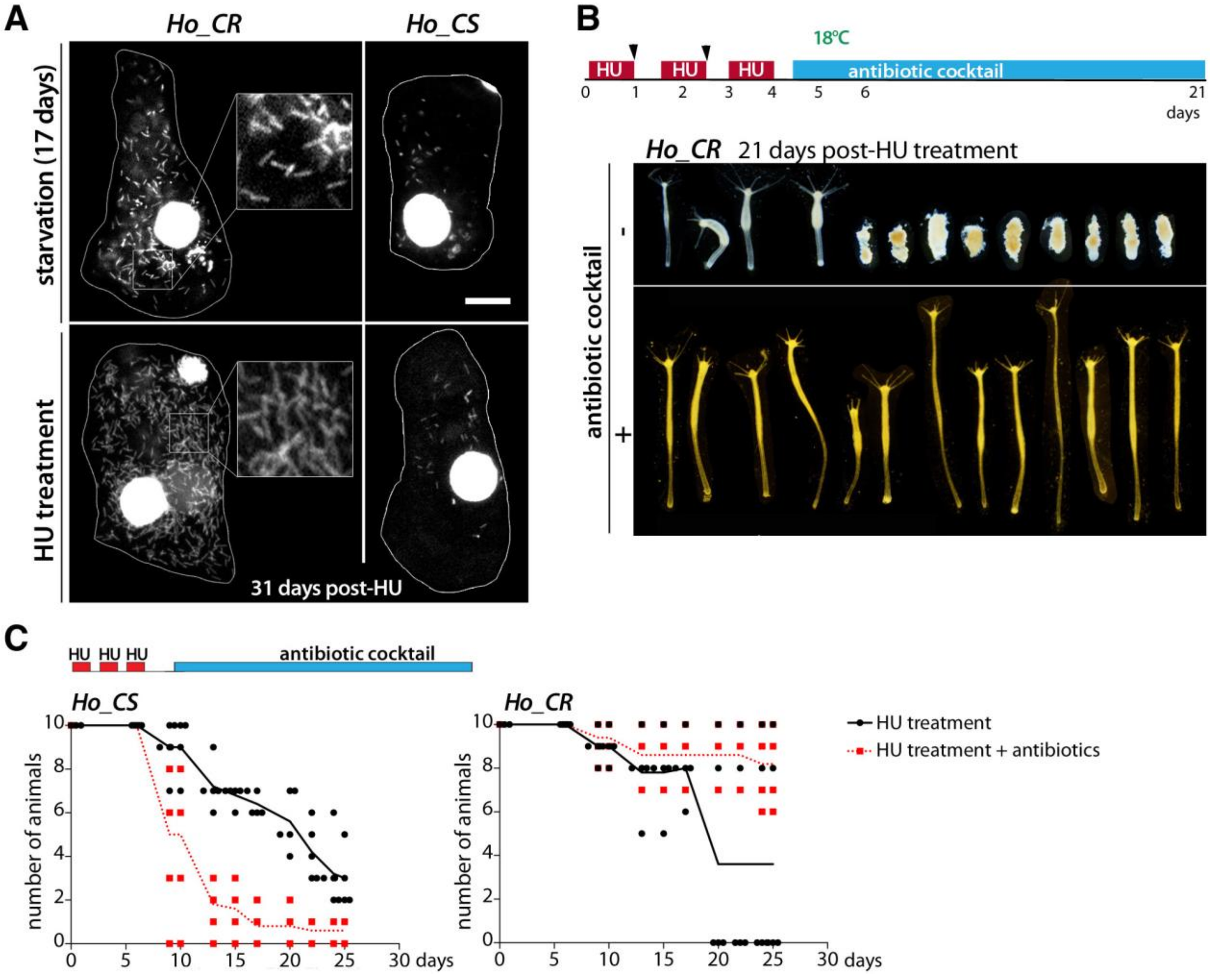

Figure S3: Distinct bacterial loads in *Ho_CR* and *Ho_CS* epithelial cells

(A) Abundance of commensal intra-epithelial bacteria in epithelial cells of *Ho_CS* and *Ho_CR* either starved for 17 days (upper row) or treated with HU as indicated in B and pictured 31 days later (lower row). Bacteria are visualized by DAPI staining. Scale bar: 10 µm. **(B)** Animal morphologies of *Ho_CR* cohorts treated with HU and subsequently exposed or not to a cocktail of antibiotics. **(C)** Survival rate of 5 cohorts of 10 HU-treated animals exposed or not to a cocktail of antibiotics. The antibiotic treatment is toxic for *Ho_CS* animals while improving the survival rate of *Ho_CR* ones.

Development • Supplementary information

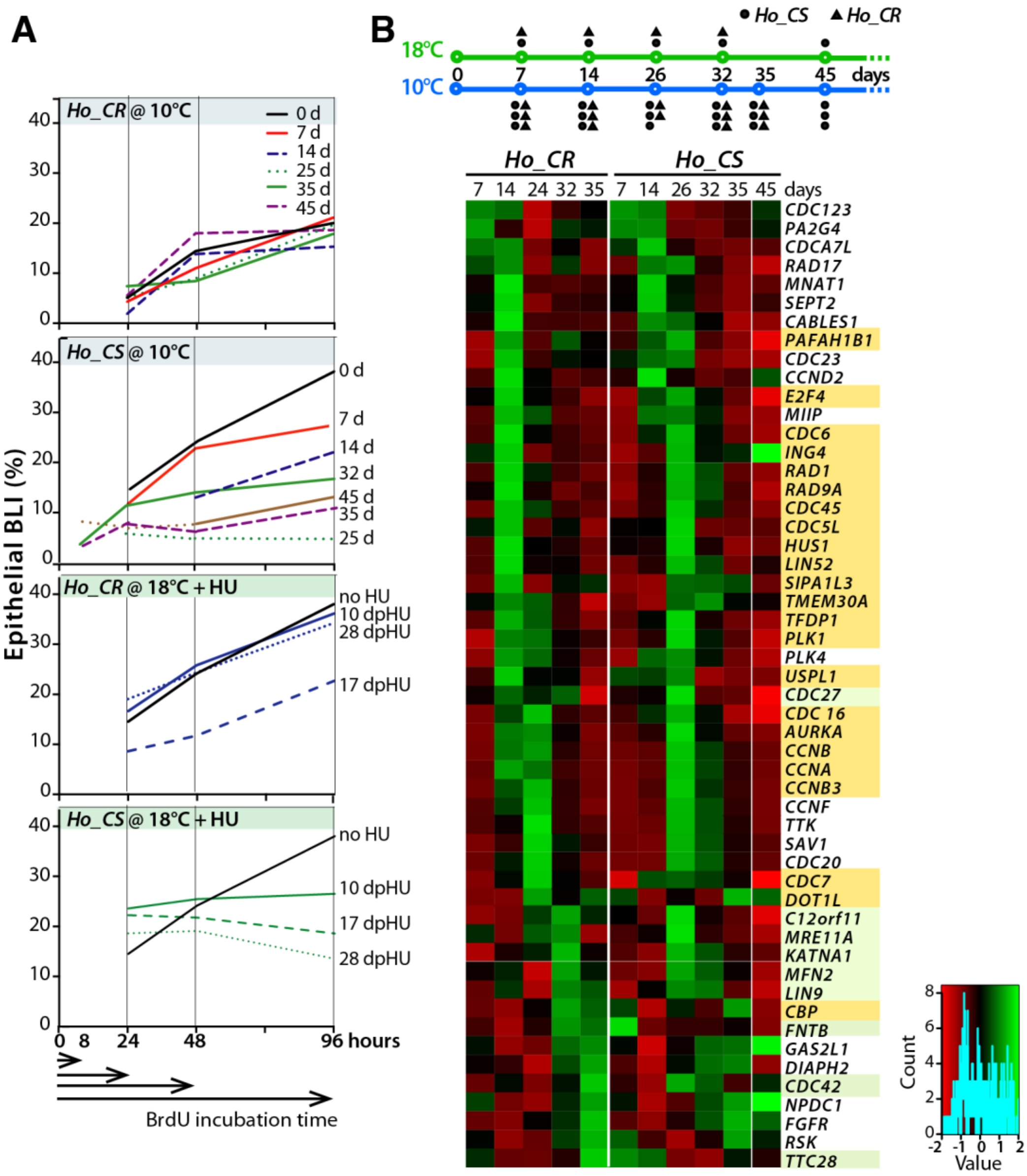

Figure S4: Comparative analysis of epithelial proliferation in *Ho_CS* and *Ho_CR* animals.

(A) Cycling activity of ESC in *Ho_CS* and *Ho_CR* animals transferred to 10°C (two upper graphs) or maintained at 18°C after HU treatment (two lower graphs). The BrdU-labeling index (BLI) was measured 7, 14, 25, 32, 45, 35 days (d) after transfer to 10°C, or 10, 17 or 28 days post-HU release (dpHU). For each time point, animals were exposed to BrdU for 24, 48 or 96 hours, then macerated for immunodetection. The fraction of BrdU-positive ESCs was counted to measure the linear progression of the cumulative eBLIs. The *Ho_CS* and *Ho_CR* cultures tested at 10°C were not fed at the same rhythm in the weeks preceding the transfer to 10°C, four times a week for *Ho_CS*, twice a week for *Ho_CR*, explaining the different eBLI values at day-0. This experiment was performed independently of the experiment shown in Figure 3D.

(B) Quantitative RNA-seq analysis of 52 *Hydra* genes orthologous to human genes annotated as involved in "cell cycle" or "cell proliferation" (www.uniprot.org, Table-S2). The experimental RNA-seq procedure is that described in Figure S2A. The heatmap shows relative fold changes defined as the ratio between the values measured at 10°C at a given time point over the value measured at 18°C at same or similar time point in *Ho_CR* and *Ho_CS* animals. Yellow and green backgrounds highlight genes whose modulations are delayed or advanced in *Ho_CS* compared to *Ho_CR* respectively. See the individual profile of each gene in **Figure S5** and access to the corresponding sequences in **Table S2**.

Development • Supplementary information

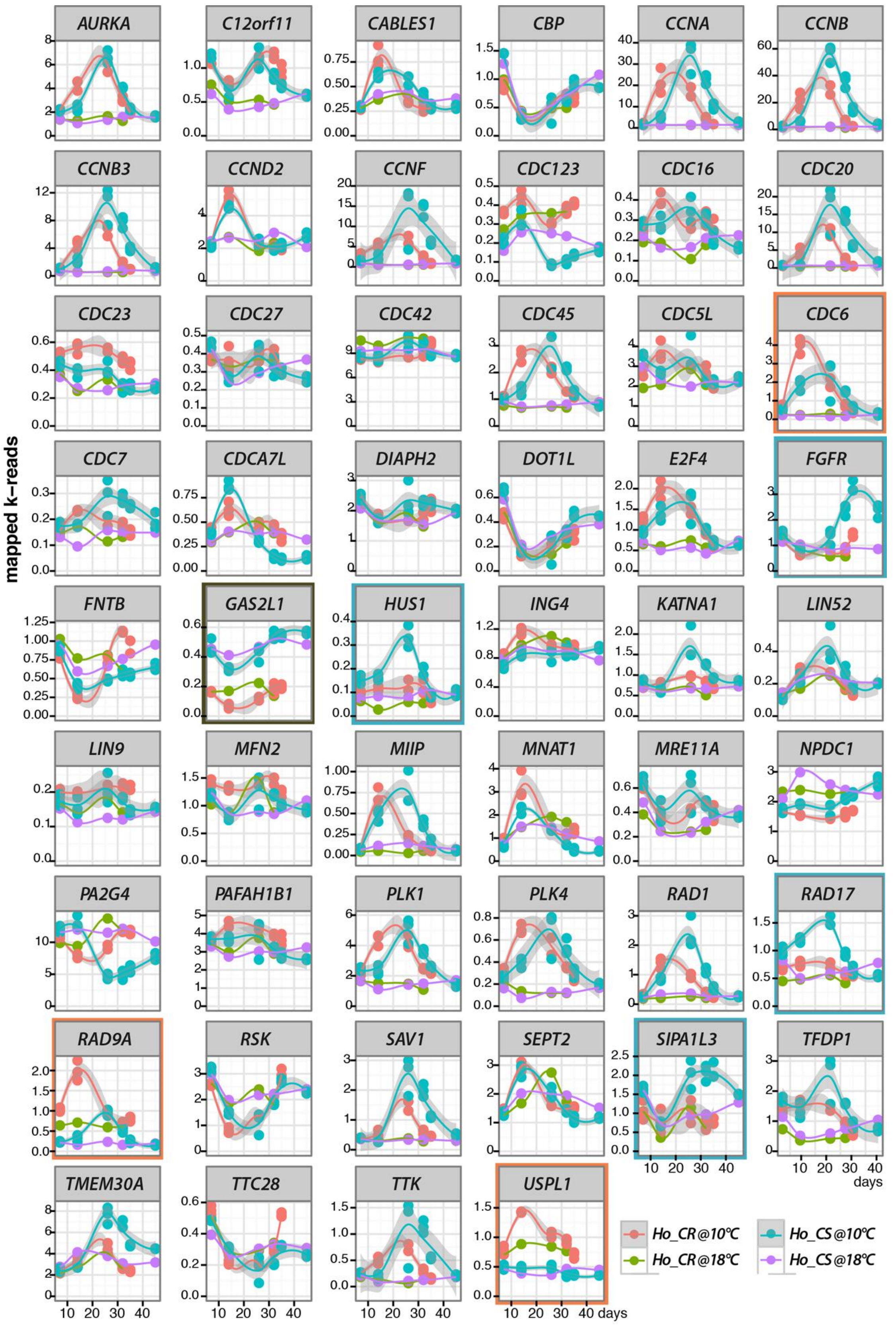

Development • Supplementary information

Figure S5: RNA-seq profiles of 52 *Hydra* orthologs to mammalian genes involved in cell cycle and/or cell proliferation

RNA-seq expression profiles of 52 cell cycle / cell proliferation genes tested in *Ho_CR* and *Ho_CS* animals maintained at 18°C or at 10°C as depicted in **Fig. S2A, S4B**. Note the delayed up-regulation of *CCNA, CCNB, CDC16, CDC45, MIIP, PLK1, PLK4, RAD1,* in *Ho_CS* when compared to *Ho_CR*. Orange frames indicate genes up-regulated in *Ho_CR* at 10°C at much higher level than in *Ho_CS (CDC6, MNAT1, RAD9A, USPL1)*, blue frames indicate genes up-regulated in *Ho_CS* at 10°C at much higher level than in *Ho_CR (CCNF, CDC20, FGFR, HUS1, KATNA1, LIN52, RAD17, SAV1, SIPA1L3, TFDP1, TTK)*, black frames indicate genes that exhibit a constitutively sustained up-regulation in *Ho_CS* when compared to *Ho_CR (GAS2L1)*. Values on x axis = days, on y axis = mapped k-reads. For access to the corresponding sequences, see **Table S2**.

Development • Supplementary information

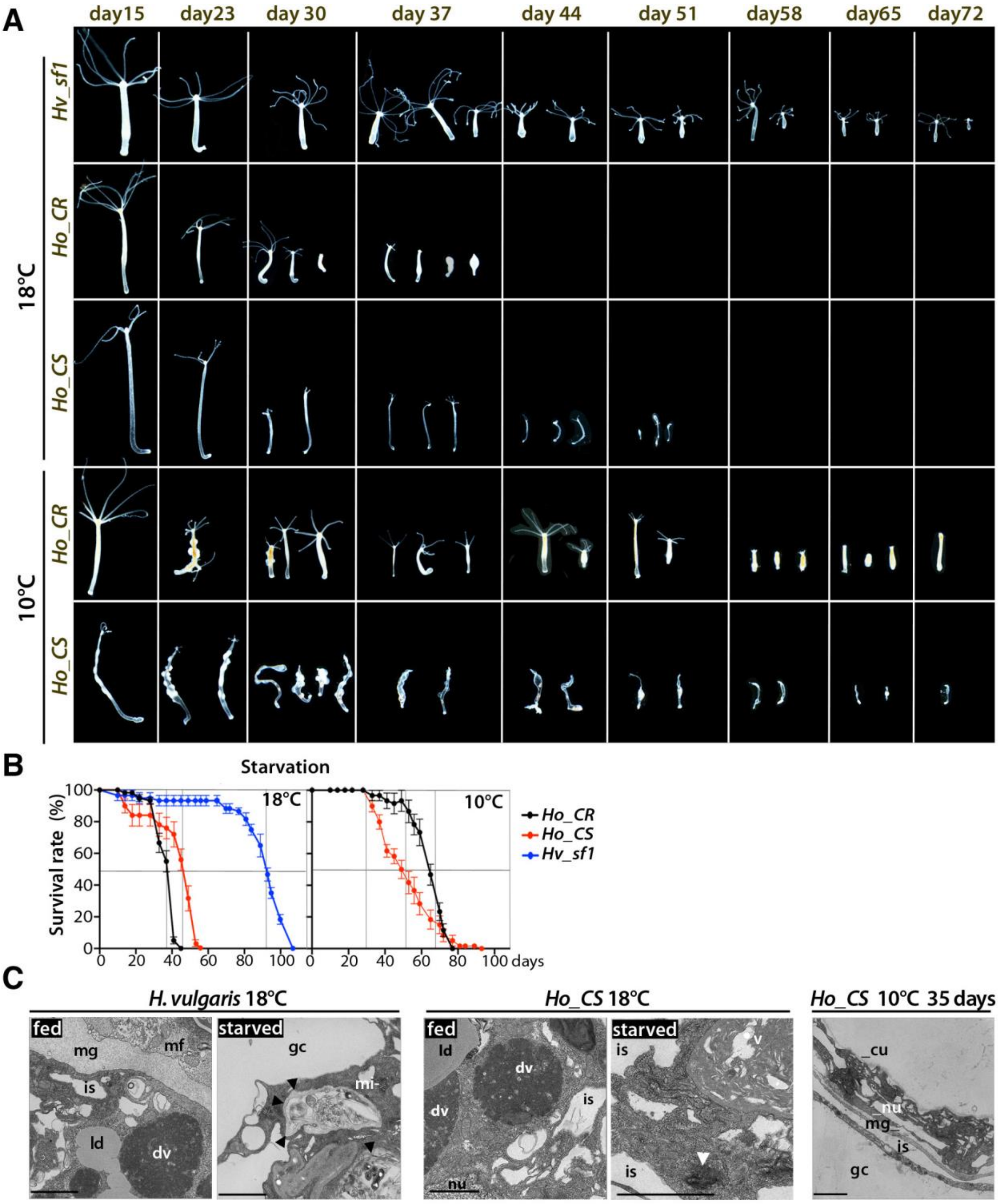
A
day15
day23
day 30
day 37
day 44
day 51
day58
day65
day72
Hv_sf1
Ho_CR
Ho_CS
18°C
Ho_CR
Ho_CS
10°C
B
Starvation
Survival rate (%)
18°C
10°C
Ho_CR
Ho_CS
Hv_sf1
days
C
H. vulgaris 18°C
Ho_CS 18°C
Ho_CS 10°C 35 days
fed
starved
mg
mf
is
ld
dv
gc
mi
nu
v
cu

Figure S6: Starvation-induced phenotypes in *Ho_CR*, *Ho_CS* and *H. vulgaris* animals

(A, B) Morphological alterations (A) and survival rates (B) recorded in starved *Hv_sf1, Ho_CR, Ho_CS* animals maintained at 18°C or 10°C (for each condition n = 6x 10 animals). At 18°C *Ho_CR* animals die by day-40 without showing morphological alterations typical of aging, while *Ho_CS* animals commonly die later, by day-58, but exhibit aging-like morphological alterations from day-30. Note that *Hv_sf1* animals resist about 50 days longer to starvation than *Ho_CS* and *Ho_CR* animals. At 10°C, starved *Ho_CR* animals undergo spermatogenesis and maintain their physiological fitness up to day-51, while starved *Ho_CS* animals exhibit aging signs from day-15, similar to those observed in animals fed twice a week (see **Figure 1D**). The two *Ho* strains exhibit a similar resistance to starvation, enhanced in case of *Ho_CR* animals maintained at 10°C when compared to18°C. **(C)** TEM views of body column sections from *Hv* (Basel strain) and *Ho_CS* animals either maintained at 18°C regularly fed or starved for 11 days, or maintained at 10°C for 35 days. Black arrowheads: autophagosome, white arrowhead: aggregate. Note the dramatically reduced gastrodermis after 35 days at 10°C in *Ho_CS* animals. Abbreviations: cu: cuticle, dv: digestive vacuole, gc: gastric cavity, is: intracellular space, ld: lipid droplets, mf: myofibril, mg: mesoglea, mi: mitochondria, nu: nucleus. Scale bars = 2 µm.

Development • Supplementary information

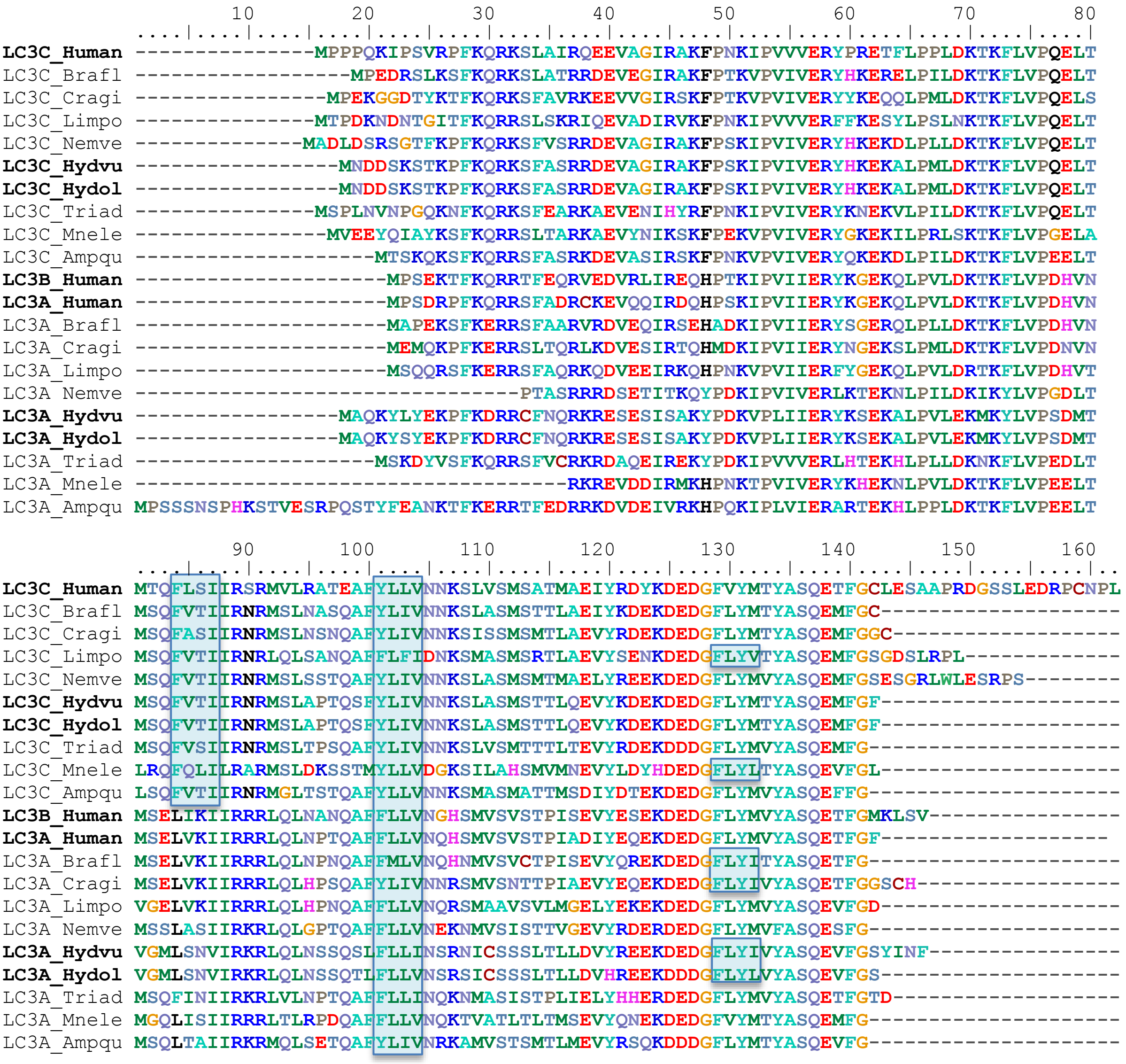

Figure S7: Alignment of the metazoan LC3/ATG8 protein sequences

The blue boxes indicate the LIR motifs with the core consensus sequence: [W/F/Y]xx[L/I/V] (Birgisdottir et al., 2013). Species abbreviations and accession numbers: **Ampqu:** *Amphimedon queenslandica* (demosponge, Porifera), LC3A: XM_003385475.2, LC3C: XM_003385524.2 (NCBI); **Brafl:** *Branchiostoma floridae* (amphioxus, Cephalochordata), LC3A: XM_002612378.1, LC3C: XM_002596383.1 (NCBI); **Cragi:** *Crassostrea gigas* (oyster, Mollusca), LC3A: XM_011449392.1, LC3C: XM_011417532.1 (NCBI); **Human**: LC3A: Q9H492, LC3B: Q9GZQ8, LC3C: Q9BXW4 (Uniprot); **Hydol:** *Hydra oligactis* (Cnidaria), LC3A: S043022c1g3_i01, R033468c0g1_i01, LC3C: S040689c0g1_i01, R036327c0g1_i01; **Hydvu**: *Hydra vulgaris* (Cnidaria), LC3A: seq54452, LC3C: c26188_g3_i03, T2M644 (Uniprot); **Limpo**: *Limulus polyphemus* (horsehoe crab, Arthropoda), LC3A: XM_013930807.1, LC3C: XM_013919901.1 (NCBI); **Mnele**: *Mnemiopsis leidyi* (combjelly, Ctenophora), LC3A: ML1904, LC3C: ML0233 (found in genome from compagen); **Nemve**: *Nematostella vectensis* (sea anemone, Cnidaria), LC3A: XM_001627787.1, LC3C: XM_001635074.1 (NCBI); **Triad:** *Trichoplax adhaerens* (Placozoa), LC3A: XM_002108002.1, LC3C: XM_002113115.1 (NCBI). See accession numbers of *Hydra* sequences in **Table S3** and sequences on HydrATLAS.

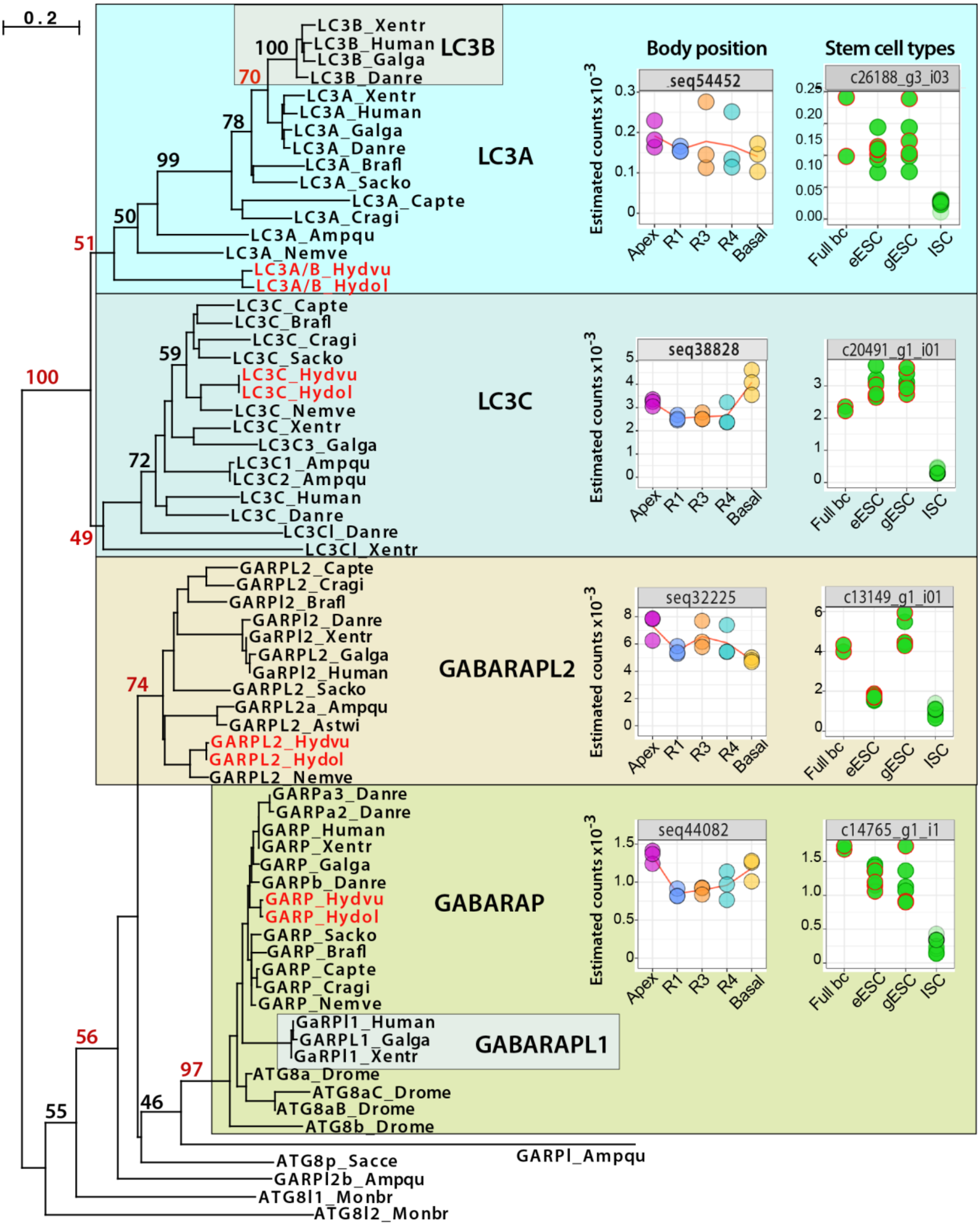

Figure S8: Phylogenetic analysis of the metazoan LC3/ATG8 gene families and RNA-seq profiles of the four *H. vulgaris LC3*-related genes

Phylogenetic tree of the LC3/ATG8 protein sequences aligned with MUSCLE and built with PhyML 3.0, tested with 100 bootstraps. *Hydra* sequences are written red. Species code is as follows: *Ampqu: Amphimedon queenslandica* (demo-sponge)*; Capte: Capitella teleta* (polychaete worm)*; Cragi: Crassostrea gigas* (oyster)*; Danre: Danio rerio* (zebrafish); *Drome: Drosophila melanogaster* (fruitfly)*; Galga: Gallus gallus* (chick)*; Hydvu: Hydra vulgaris; Hydol: Hydra oligactis; Monbr: Monosiga brevicollis* (choanoflagellate)*; Nemve: Nematostella vectensis* (sea anemone); *Sacce: Saccharomyces cerevisiae* (yeast)*; Sacko: Saccoglossus kowalesvskii* (acorn worm)*; Xentr: Xenopus tropicalis* (Western clawed frog). The four main families MAP1LC3A (LC3A), MAP1LC3C (LC3C), GABARAPL2 (GARPL2) and GABARAP (GARP) include sequences from deuterostomes,

protostomes, cnidarians and poriferans. The GARPl_Ampqu sequence appears related to GABARAP although highly derived, while the two families LC3B and GABARAPL1 are vertebrate-specific duplications of LC3A and GABARAP respectively. Note the *Drosophila* sequences that all cluster in the GABARAP family. By contrast the non-metazoan sequences from yeast or choanoflagellates do not cluster in any of these four metazoan families. The graphs on the right show the RNA-seq profiles of the four LC3/ATG8 family members expressed in homeostatic *H. vulgaris*, along the body column (bc, left) and in each stem cell populations (right) as reported in ref. (Wenger et al., 2016; Wenger et al., 2019). Abbreviations: R1: upper body column, R3: upper mid-gastric region, R4: lower mid-gastric region, Foot: peduncle and basal disk; eESC: epidermal epithelial stem cells; gESC: gastrodermal epithelial stem cells; ISC: interstitial stem cells.

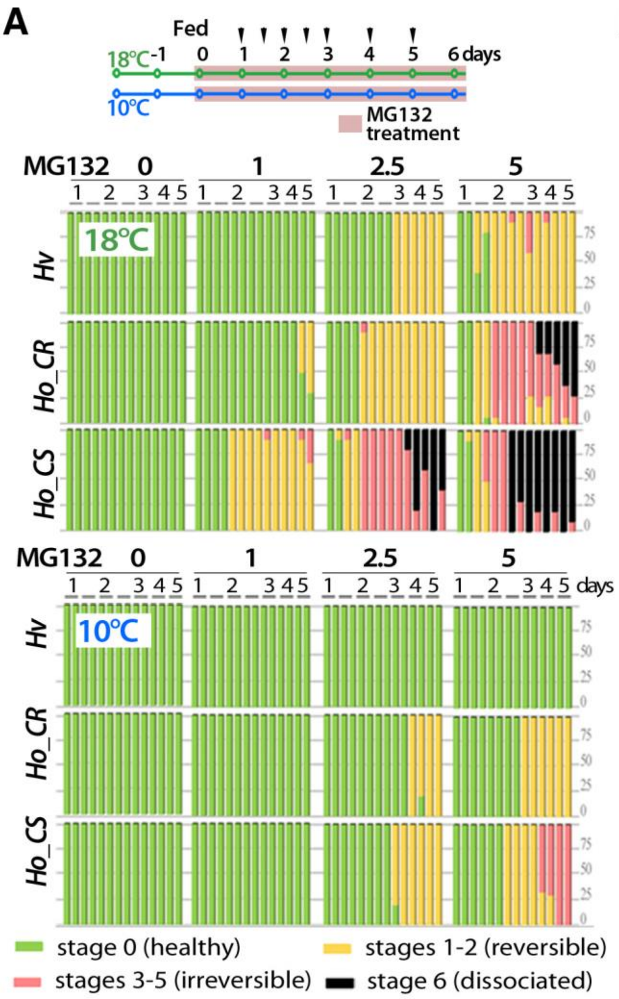

Figure S9: Different sensitivity to MG132 in *Ho_CS, Ho_CR* and *Hv.*

(A) Toxicity recorded in animals (n= 2x 10 /strain) maintained at 18°C (top) or 10°C (bottom) and continuously exposed to the proteasome inhibitor MG132 at indicated concentrations (0, 1, 2.5 or 5 µM) for 1, 2, 3, 4 or 5 days. **(B)** Resistance to proteasome inhibition tested in *Ho_CS* and *Ho_CR* animals exposed to MG132 (5 µM) for 16 hours and then pictured live. When maintained at 18°C, animals were either fed 4x a week or starved for 14 days, at 10°C animals were fed twice a week. Note the higher sensitivity of *Ho_CS* animals that rapidly exhibit shortened, “ball-shaped” tentacles (arrows) as signs of stress.

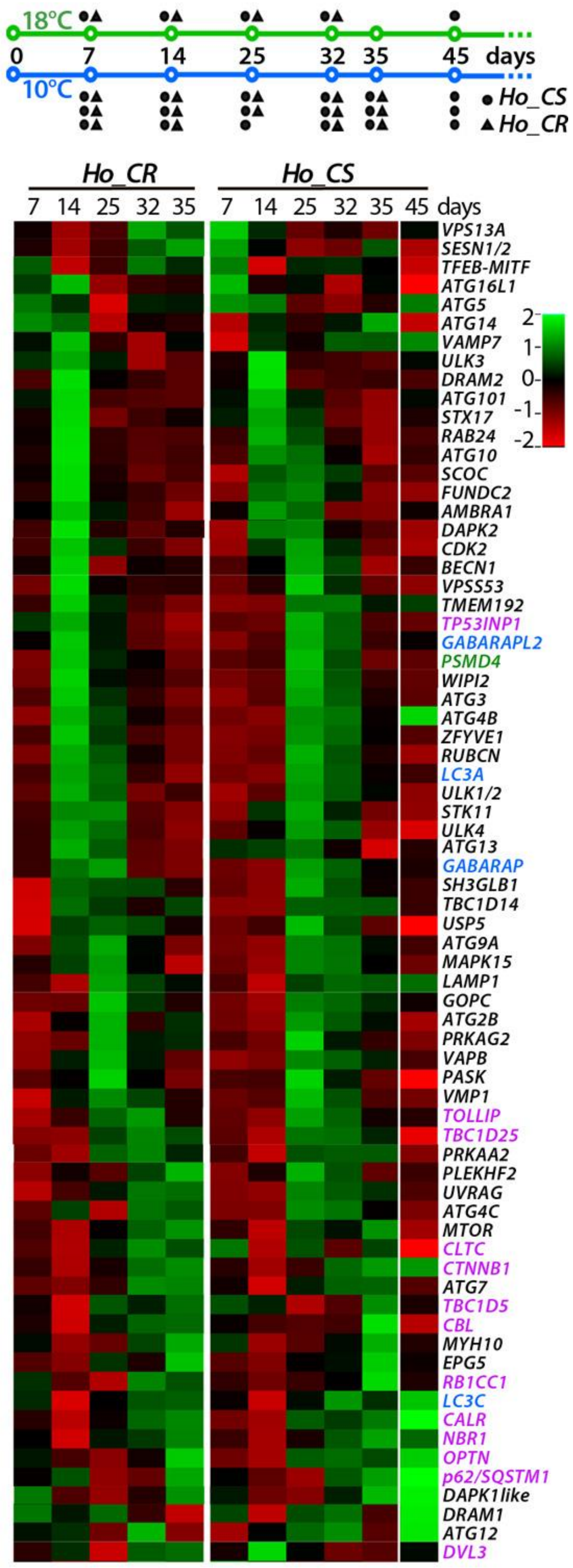

Development • Supplementary information

Figure S10: Comparative transcriptomic analysis of 75 *Hydra* orthologs to mammalian autophagy genes in *Ho_CS* and *Ho_CR*

Upper scheme: Experimental design of the quantitative RNA-seq analysis. RNAs from *Ho_CR* and *Ho_CS* animals were prepared at indicated time points with biological triplicates for animals maintained at 10°C. Lower panel: Heatmap showing the log2 fold changes of RNA-seq levels of 75 *Hydra* genes orthologous to human genes involved in autophagy. Fold changes are defined as ratio between the values measured at 10°C at a given time point over the value measured at 18°C at same or similar time point in a given strain. For technical details, see the Methods section. Gene names written **black** encode regulators of autophagy initiation and progression, **purple**: autophagy receptors or adaptors interacting with LC3/ATG8, **blue**: members of the LC3-GABARAP family, **green**: proteasome components. See the corresponding individual expression profiles in **Figure S11** and access to corresponding sequences in **Table-S3.** Note in *Ho_CS* the delayed activation of most autophagy genes and the late up-regulation of *NBR1* and *p62/SQSTM1*.

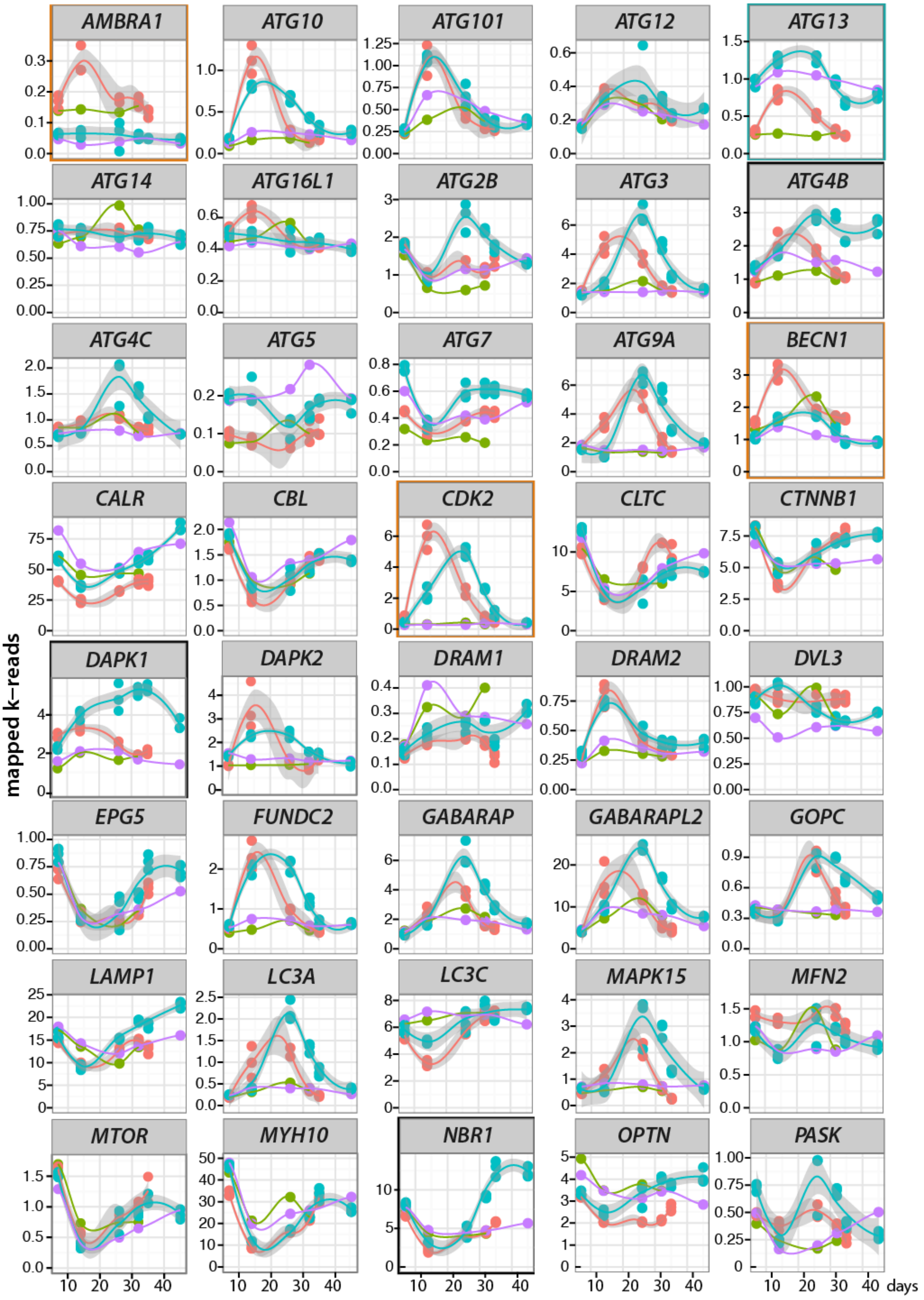

Development • Supplementary information

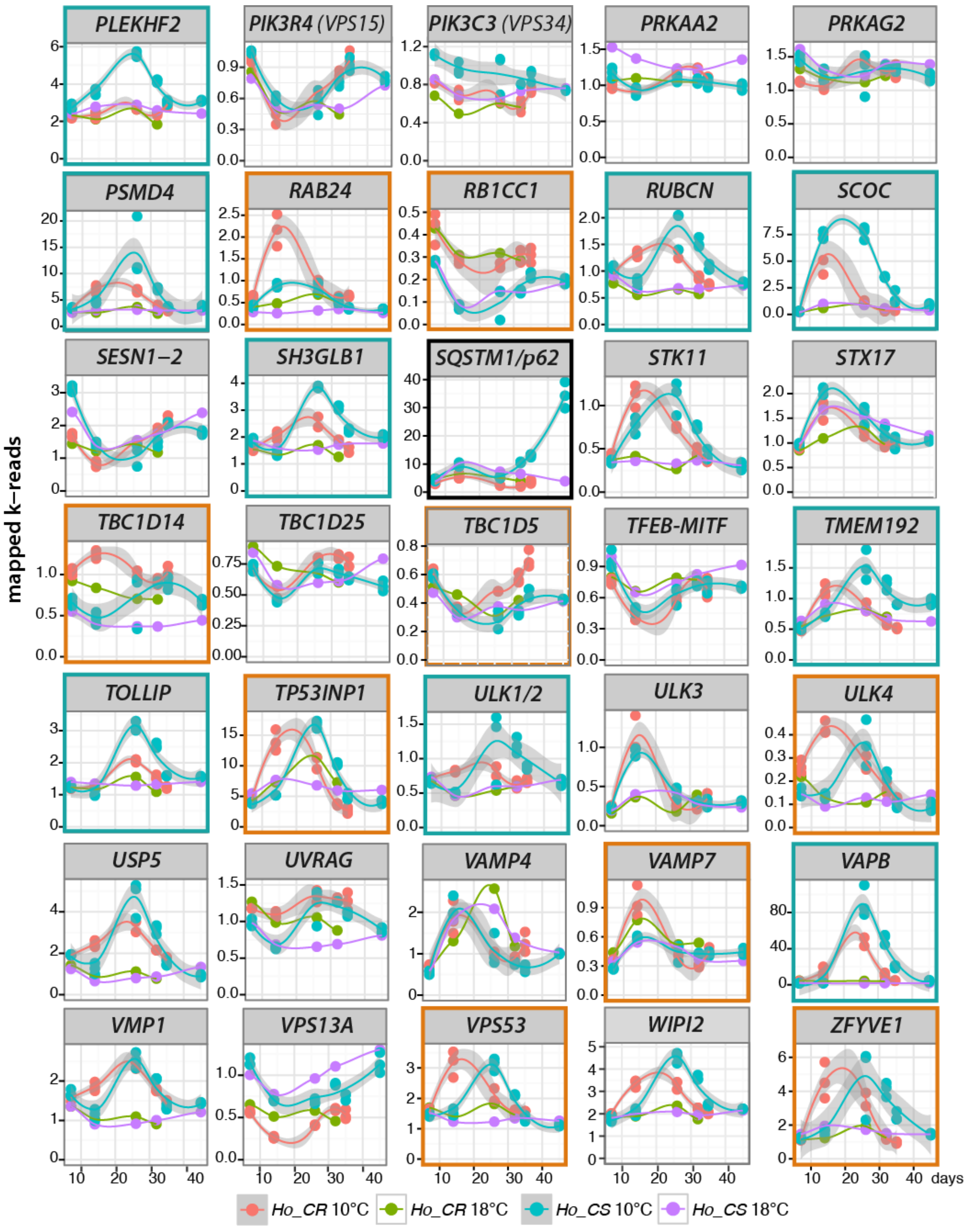

**21/51 genes transiently up-regulated in both strains but delayed by 10 days in *Ho_CS*** (10/20 exhibit higher levels in *Ho_CS* – underlined -)	*ATG3, ATG9A, ATG13, CDK2, DAPK2, GABARAP, GABARAPL2, LC3A, PSMD4, RUBCN, SCOC, SH3GLB1, STK11, TBC1D14, TMEM192, TP53INP1, ULK1/2, ULK4, VPS53, WIPI2, ZFYVE1*
**14/51 genes similarly transiently up-regulated in *Ho_CS* and *Ho_CR*,** peaking at day14 or day25, (underlined: exhibit higher levels in *Ho_CS)*	*ATG10, ATG101, ATG12, ATG9A, DRAM2, FUNDC2, GOPC, MAPK15, STX17, TOLLIP, ULK3, USP5, VAMP4, VAPB*
**5/51 up-regulated in *Ho_CR*** but poorly in *Ho_CS*	*AMBRA1, ATG16L1, BECN1, RAB24, VAMP7*
**4/51 up-regulated in *Ho_CS*,** not or poorly in *Ho_CR*	*ATG2B, ATG4C, PLEKHF2, TOLLIP*
**7/51 genes sustainably up-regulated in *Ho_CS*,** *i.e.* showing a temporal accumulation	*ATG4B, ATG7, CALRC, DAPK1, LAMP1, NBR1, p62/SQSTM1*

Development • Supplementary information

Figure S11: RNA-seq profiles of 75 *Hydra* orthologs to mammalian autophagy genes

RNA-seq expression profiles of 75 autophagy genes tested in *Ho_CR* and *Ho_CS* animals maintained at 18°C or at 10°C as depicted in **Figure_S10**. Orange frames indicate genes up-regulated in *Ho_CR* at 10°C but not at all or less in *Ho_CS*, blue frames indicate genes up-regulated in *Ho_CS* at 10°C but not at all or less in *Ho_CR*, black frames indicate genes that exhibit a sustained up-regulation at late time-points in *Ho_CS* but not in *Ho_CR*. Values on x axis = days, on y axis = mapped k-reads. For the corresponding sequences, see **Table S3**.

Development • Supplementary information

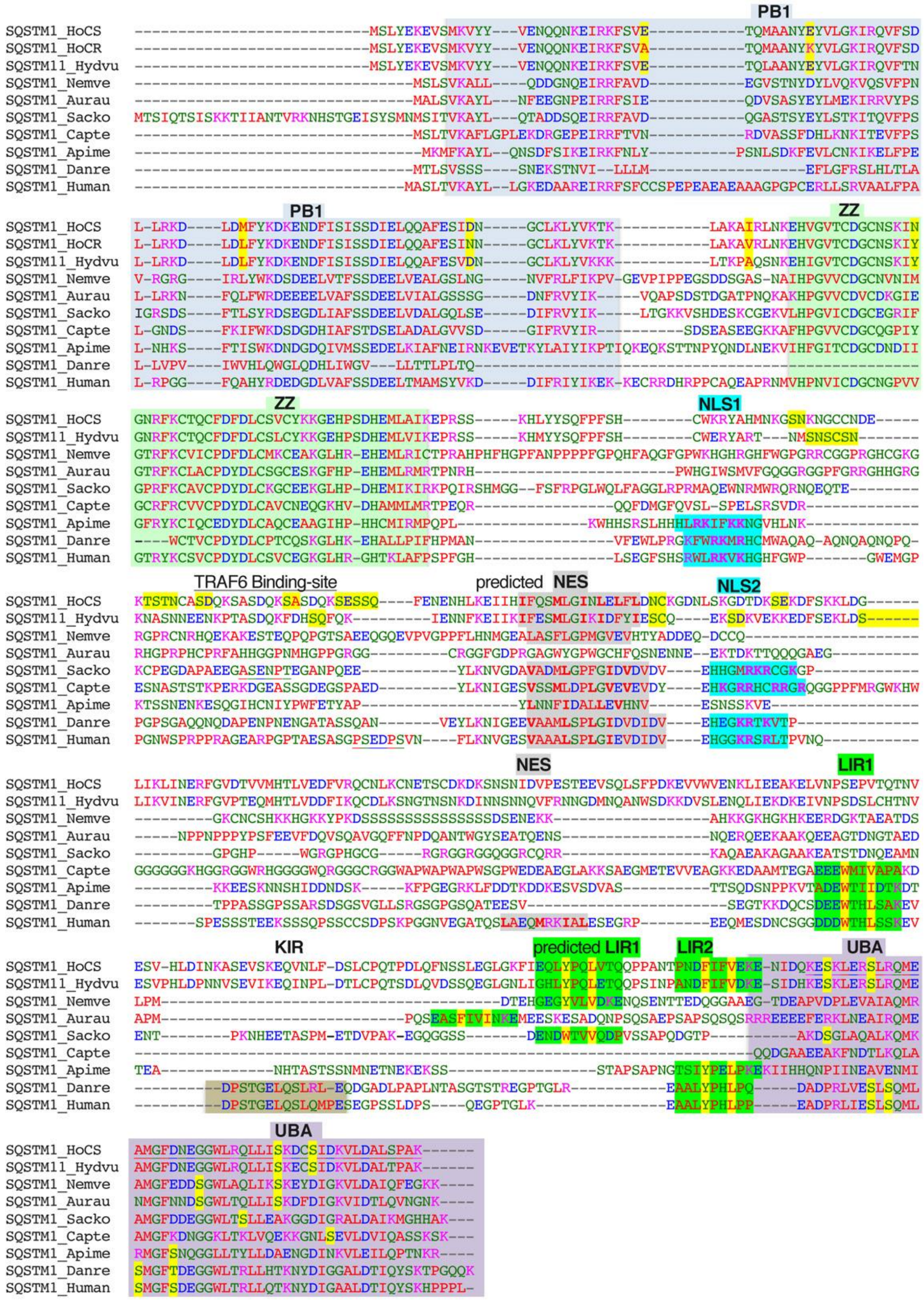

Development • Supplementary information

Figure S12: Alignment of vertebrate and non-vertebrate p62/SQSTM1 protein sequences

The alignment was obtained on MUSCLE (www.ebi.ac.uk/Tools/msa/muscle/) and manually corrected to align the functional domains as listed in refs (Seibenhener et al., 2004, Birgisdottir et al., 2013, Bitto et al., 2014): **PB1**, Phox and Bem1 domains (blue) involved in protein kinase binding; **ZZ**, ZZ-type zinc finger domain (green); **NLS1** and **NLS2,** nuclear localization signals 1 and 2 (turquoise); **NES,** nuclear export signal (grey); **LIR**, LC3- interacting region (green-yellow); **KIR,** KEAP-interacting region (beige); **UBA**, ubiquitin-associated domain (purple). In non-vertebrate sequences, the putative LIR, NLS and NES motifs were manually identified following the consensus sequence reported in refs (Pankiv et al., 2007) and (Birgisdottir et al., 2013): LIR = x_{-5}(s) x_{-4}(dt) x_{-3}(desg) x_{-2}(ds) [WFY] x_1(evtd) x_2(implt) [LIV] x_4(pdsr) x_5 ; NLS = [R] [K] x_1(vs) [K] or [K] [R] x_1(vs) [R]; NES = [L] x_1 x_2 x_3 (2, 3) [LIVFM] x_5 x_6 (2 or 3) [LI] x_7 [LI]. The UBA sequence used for raising the anti-*Hydra* p62/SQSTM1 antibody is underlined (KESKLER ....ALSPAK). Species code and accession numbers are given in **Figure S13A** and **Table S3**.

Development • Supplementary information

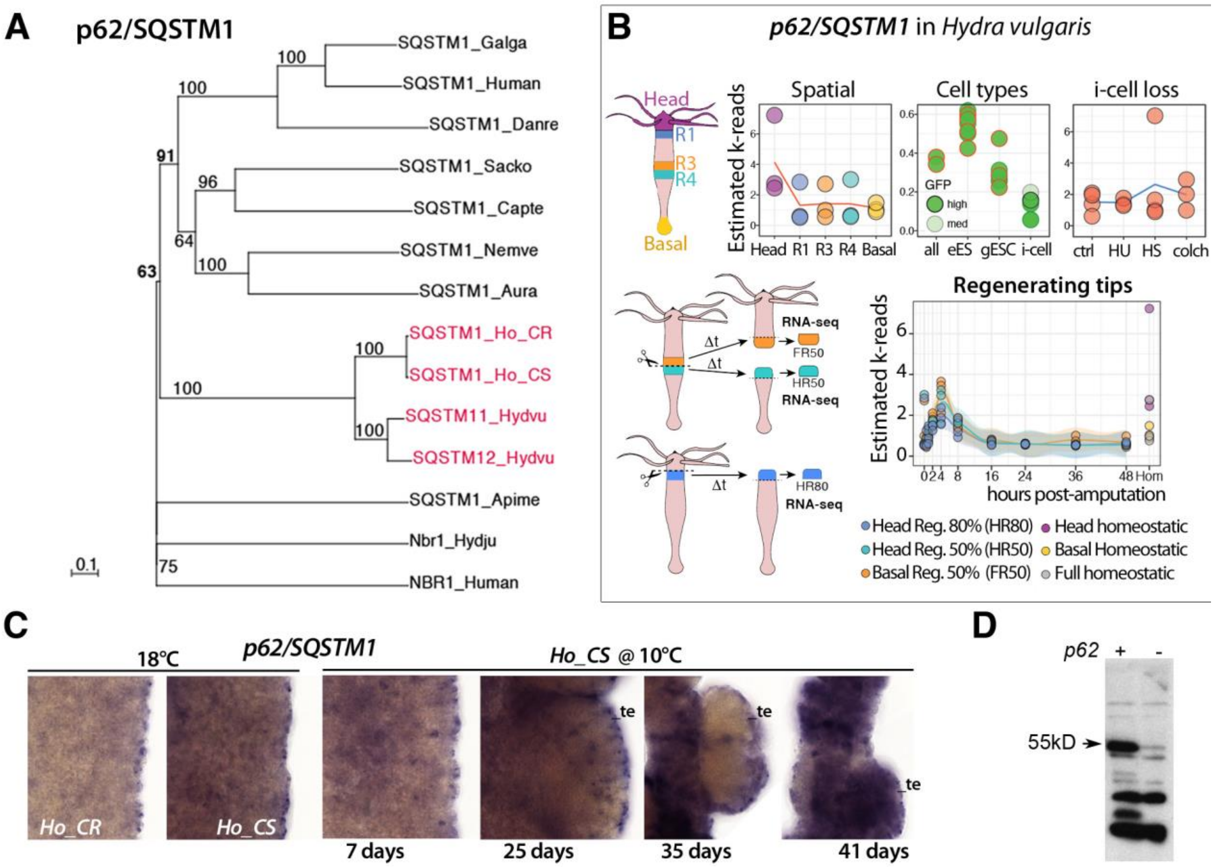

Figure S13: Phylogenetic tree and expression analysis of *p62/SQSTM1* in *Hydra*

(A) Phylogenetic tree of p62/SQSTM1 protein sequences aligned with MUSCLE and built with PhyML 3.0, tested with 500 bootstraps. NBR1 sequences were used as outgroup. Species code and sequence accession numbers are for *Apime: Apis mellifera* (XP_392222.3); *Aurau: Aurelia aurita (*Q5EN85); *Capte: Capitella teleta* (gb|ELT88176.1); *Danre: Danio rerio* (Q6NWE4); *Galga: Gallus gallus* (F1NA86); *Human*: Q13501; *Ho_CS, Ho_CR: Hydra oligactis* (see **Table-S3**); *Hydvu: Hydra vulgaris* (XP_004206050.1; T2MDZ6); *Nemve: Nematostella vectensis* (A7RN64); *Sacko: Saccoglossus kowalevskii* (XP_002737931.1). **(B)** RNA-seq profiles of *H. vulgaris p62/SQSTM1* as reported in (Wenger et al., 2014, Wenger et al., 2016, Wenger et al., 2019). **Body position**: expression measured at 5 distinct levels along the body axis of *H. vulgaris* Jussy strain; **Stem cell types:** expression measured in the three stem cell populations of *H. vulgaris* AEP (after FACS sorting cells of transgenic strains that constitutively GFP in one or the other cell type); **i-cell loss:** expression measured 10 days after the heat-shock or drug-induced elimination of cycling interstitial cells; **Regeneration**: expression measured in regenerating tips at 9 time points of three distinct regenerative processes in *H. vulgaris* Jussy strain (HR50, FR50: head or foot regeneration after mid-gastric bisection; HR80: head regeneration after decapitation). **(C)** Whole-mount *in situ* hybridization showing an ubiquitous expression of *p62/SQSTM1* in *Ho_CR* and *Ho_CS* at 18°C, progressively enhanced in epithelial cells of *Ho_CS* animals undergoing aging. **(D)** Testing of the anti-Hydra p62/SQSTM1 antisera (batch 507) against the *Hydra* p62/SQSTM1 protein expressed in TNT-coupled reticulocyte lysate (Promega) (lane +); the empty vector was used as negative control (lane -). The expected weight of *Ho_CS* p62/SQSTM1 is 54.53 kD.

Development • Supplementary information

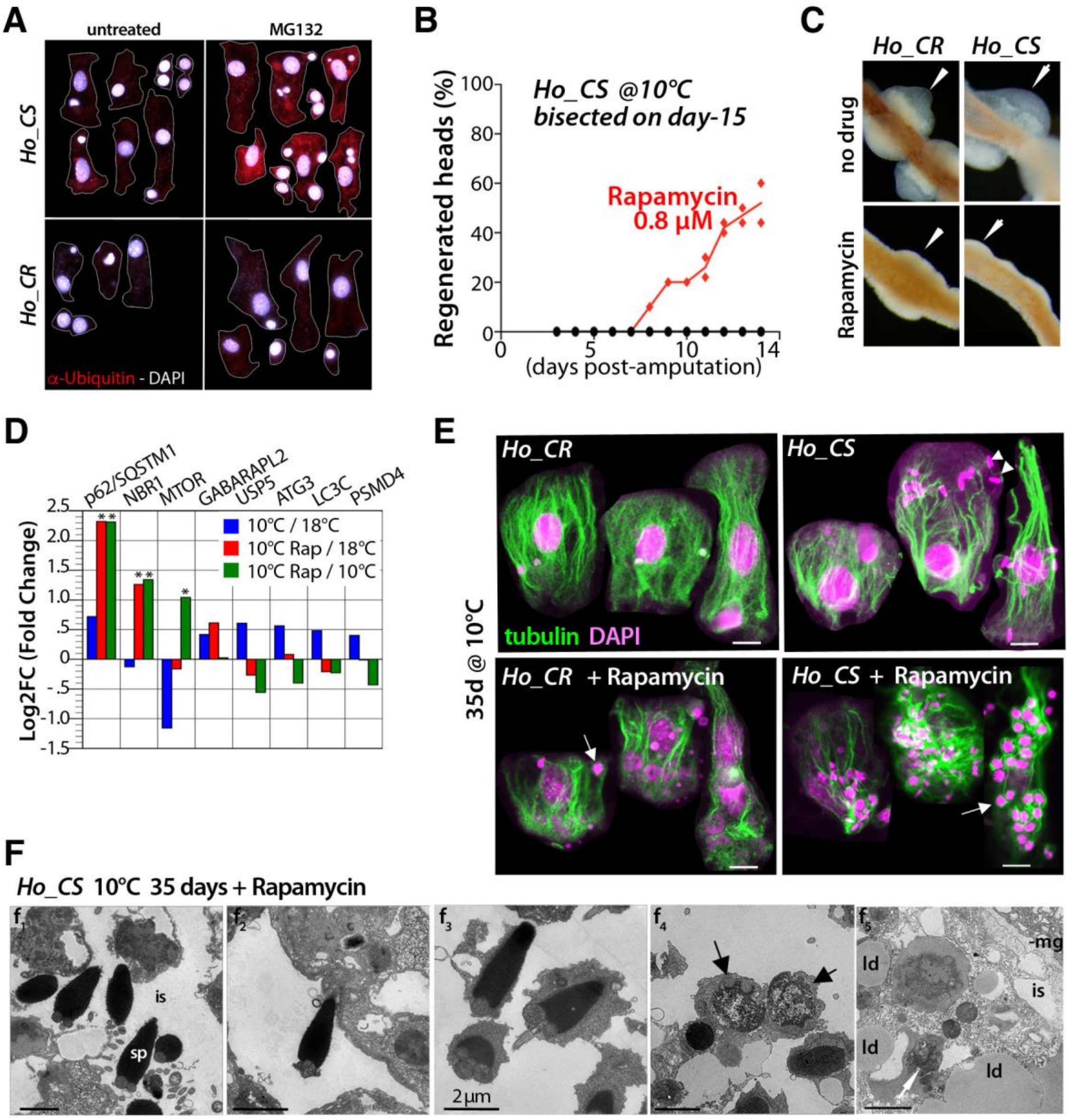

Figure S14: Anti-aging role of rapamycin in *Ho_CS Hydra*

(A) Immunodetection of ubiquitin in cells from *Ho_CS* and *Ho_CR* animals maintained at 18°C and exposed or not to MG132 for 16 hours. **(B)** A continuous exposure to rapamycin from day-2 after transfer to 10°C efficiency rescues head regeneration in *Ho_CS* bisected on day-15. **(C)** Testes (arrowheads) exhibit a reduced size in animals continuously exposed to rapamycin. **(D)** Proteomic analysis performed on *Ho_CS* animals maintained for 35 days either at 18°C or at 10°C where they were exposed or not to rapamycin for 32 days, *: 0.05, **: 0.001 significance. **(E)** Engulfed cells detected with an anti a-tubulin antibody (green) and DAPI staining (pink) in epithelial cells from *Ho_CS* and *Ho_CR* animals fixed after 36 days at 10°C. Arrows: nuclei from immature germ cells; arrowheads: sperm cell nuclei. **(F)** Sperm cells (sp) engulfed in epithelial cells of rapamycin-treated *Ho_CS* animals taken at 35 dpt. Sperm cells can be detected in the intracellular space (is, f1, f2), surrounded by cytoplasm (f3) and digested (f4, f5). Black arrows: mitochondria at the base of sperm cells. Abbrevations: is: intracellular space, ld: lipid droplet, mg: mesoglea. Scale bars = 2 μm.

Development • Supplementary information

```
                                                          WD40 repeat 1
WIPI2_HoCS    ----------------------MNLAANGADP-SGDLLFTNFNQDKT------------------SLAVGTRLGYKLFSLNSV
WIPI2_HoCR    ----------------------MNLAANGADP-SGDLLFTNFNQDKT------------------SLAVGTRLGYKLFSLNSV
WIPI2_Hydvu   ----------------------MNLAANGADP-SGDLLFTNFNQDKT------------------SLAVGTRLGYKLFSLNSV
WIPI2_Nemve   ----------------------MNLAAYGGDP-TGNLLFVNFNQDCT------------------SLAIGTKSGYKLYSLGSV
WIPI2_Sacko   ----------------------MNLASHAGEA-NANLLFVNVNQDCT------------------SLAVGAKSGCRLFSLGSV
WIPI2_Capte   ----------------------MNLASQGGDP-NNDLIFVNFNQDCS------------------SLAVGSKHGYKLFSLNSA
WIPI2_Apime   MQNLVHNVDVLTQGLKYSISGLMNLANQTTDP-QSRVFFVNFNQDCT------------------SLAVGSKSGYKLFSLVSV
WIPI2_Danre   ----------------------MNLASQSGEAGCSQLLFANFNQDNT------------------SLAVGTKSGYKFFSLSSV
WIPI2_Human   ----------------------MNLASQSGEAGAGQLLFANFNQDNTEVKGASRAAGLGRRAVVWSLAVGSKSGYKFFSLSSV
                         WD40 repeat 2                    WD40 repeat 3    ATG16 binding
WIPI2_HoCS    EKLDEIHH-YDKGDVCIVERLFSSSLVAIVSLSAPRKLKVCHFKKGTEICNYSYPNTILAVRLNRVRLLVVLEESLYIHNIRD
WIPI2_HoCR    EKLDEIHH-YDKGDVCIVERLFSSSLVAIVSLSAPRKLKVCHFKKGTEICNYSYPNTILAVRLNRVRLLVVLEESLYIHNIRD
WIPI2_Hydvu   EKLDEIHH-YDKGDVCIVERLFSSSLVAIVSLSAPRKLKVCHFKKGTEICNYSYPNTILAVRLNRVRLLVVLEESLYIHNIRD
WIPI2_Nemve   EKLEEIYEYGGTPDICIVERLFSSSLVAIVSLSAPRKLKVCHFKKGTEICNYSYPNTILAVRLNRVRLLVVLEESLYIHNIRD
WIPI2_Sacko   DKLEAIYEHNETEDICIVERLFSSSLVAMVSLSSPRKLKVCHFKKGTEICNYSYPNTILAVRLNRLRLIVALEESLYIHNIRD
WIPI2_Capte   DKLENIYE-NDTEDICTVERLFSSSLVAIVGLSSPRKLKVCHFKKGTEICNYSYSNTILAVRLNRLRLVVCLEESLYIHNIRD
WIPI2_Apime   DHLEKIYE-NDTEDIYIVERLFSSSLVAVVSLRSPRKLKVCHFRKGTEICHYSYSNTILAVKLNRARLVVCLEESLYIHNIRD
WIPI2_Danre   DKLEQIYECTDTEDVCIVERLFSSSLVAIVSLKAPRKLKVCHFKKGTEICNYSYSNTILAVKLNRQRLIVCLEESLYIHNIRD
WIPI2_Human   DKLEQIYECTDTEDVCIVERLFSSSLVAIVSLKAPRKLKVCHFKKGTEICNYSYSNTILAVKLNRQRLIVCLEESLYIHNIRD
                          WD40 repeat 4                                 WD40 repeat 5
WIPI2_HoCS    MKVLHTIRDTPPNRFGLCALSDNAENCYLAYPGNNRIGEVQIFDGINLRAVTLIAAHDAPLAAITFNIHATLLATASEKGTVI
WIPI2_HoCR    MKVLHTIRDTPPNRFGLCALSDNAENCYLAYPGNNRIGEVQIFDGINLRAVTLIAAHDAPLAAITFNIHATLLATASEKGTVI
WIPI2_Hydvu   MKVLHTIRDTPPNRFGLCALSDNAENCYLAYPGNNRIGEVQIFDGINLRAVTLIAAHDAPLAAITFNTHATLLATASEKGTVI
WIPI2_Nemve   MKVLHTIRDTPPNPSGLCALSVNSDNCYLAYPGSNQIGEVQIFDAVNLRAVTMIPAHDSPVASMAFNHMGTKLATASEKGTVI
WIPI2_Sacko   MKVLHTIRDTPPNPIGLCALSINNDNCYLAYPGSSQIGEVQIFDSVNLRAVNMIPAHDSPLAALMFNPTATKLATASEKGTVI
WIPI2_Capte   MKVLHTIRDTPPNPSGLCTLSNSNDNCFLAYPGSSQIGEVQIFDAVNLRAVTMIPAHDNPLAAMAFNSTGTRIATASEKGTVI
WIPI2_Apime   MKVLHTIRDTPPNLAGLCTLSINSDNCYLAYPGSNTIGEVQIFDAINLQAKTMIPAHDSPLAALAFSPNGTKVATASEKGTVI
WIPI2_Danre   MKVLHTIRETPPNPSGLCALSISNDNCYLAYPGSATIGEVQVFDTVNLRAANMIPAHDSPLAALAFDASGTKLATASEKGTVI
WIPI2_Human   MKVLHTIRETPPNPAGLCALSINNDNCYLAYPGSATIGEVQVFDTINLRAANMIPAHDSPLAALAFDASGTKLATASEKGTVI
                   PI3P binding     WD40 repeat 6
WIPI2_HoCS    RVFSIPDGLKLFEFRRGMKRCAQINSLAFSNDSLFLVSSSNTETVHVFKLETEKTS--KEEPSSQTWMGYFGKALMAPASYLP
WIPI2_HoCR    RVFSIPDGLKLFEFRRGMKRCAQINSLAFSNDSLFLVSSSNTETVHVFKLETEKTS--KEEPSSQTWMGYFGKALMAPASYLP
WIPI2_Hydvu   RVFSIPDGLKLFEFRRGMKRCAQINSLAFSTDSLFLASSSNTETVHVFKLETEKTI--KEEPSSQTWMGYFGKALMAPASYLP
WIPI2_Nemve   RVFSIPDGQKLYEFRRGVKRCVTINSLAFSQDSLFLSASSNTETVHIFKLEMPKD---KPQEESQGWMGYFGKAL-SPTNYLP
WIPI2_Sacko   RVFCIPEGQKLFEFRRGMKRCVSISSLAFSADSVFLSASSNTETVHIFKLETPRD---KPNEEPASWMGYVSKALMSSASYLP
WIPI2_Capte   RVFSIPDGQKMFEFRRGVKRCVTIYSLAFSPDSLFLCCSSNTETVHIFKLETVKDP--KVFEEPQGWMGYFGQALKTSANYLP
WIPI2_Apime   RVFHVHDGTKLFEFRRGVKRCVSISSLAFSVDSMFLCCSSNTETVHIFKLEEPKEALRQTAEESQTWMGYLTKAVSASANYLP
WIPI2_Danre   RVFSIPEGQKLFEFRRGVKRCVSICSLAFSMEGLYLSASSNTETVHIFKLETQRE---KPQEEPTTWTGYFGKVLMASTTYLP
WIPI2_Human   RVFSIPEGQKLFEFRRGVKRCVSICSLAFSMDGMFLSASSNTETVHIFKLETVKE---KPPEEPTTWTGYFGKVLMASTSYLP
                                  WD40 repeat 7
WIPI2_HoCS    SQMTEVFSQGRAFAIAKLPNAGQRNICALTVINKLPRILVASADGYLYIYNLDPTDGQECPILRQFSLIPSEDDVMNVPEGEN
WIPI2_HoCR    SQMTEVFSQGRAFAIAKLPNAGQRNICALTVINKLPRILVASADGYLYIYNLDPTDGQECPILRQFSLIPSEDDVMNVPEGEN
WIPI2_Hydvu   SQMTEVFSQGRAFAIAKLPNAGQRNICALAVINKLPRILVASADGYLYIYNLDPTDGQECPILRQFSLIPSEGDVMNVPEGEH
WIPI2_Nemve   SQVTEVFNQGRAFANVHLPVAGLRNVCAVATIGKLPRLLVSSADGYLYIYNIDPEDGGDCTLLKQHR----------------
WIPI2_Sacko   SQVTDVFNQGRAFAIVKLPFAGLKNICALATIQKLPRVLVASQDGYLYIYNLDPAEGGDCTLLKQHRLIGDMSCEVRETDKT-
WIPI2_Capte   SQVTEMFNQGRDFAIARLPFSGLRNVCTLTNIQKLPRLLVASQNGYLYMYNLDPMEGGECTLLKQHRLDGQLDALTAEVSPPA
WIPI2_Apime   SQVTDVFNQGRAFASVHLPFQGLKNVCAITVVHKVLRLLVASAEGYLYVYNLDSTEGGDCTLLKQHRLDGKRDEVDCASVSTA
WIPI2_Danre   AHVTEMFTQGRAFATVRLPFSGHKNICALAIIQKIPRLLVAAADGYLYLYNLDPQEGGECTLMKQHKLDGSAEPANEILEQTA
WIPI2_Human   SQVTEMFNQGRAFATVRLPFCGHKNICSLATIQKIPRLLVGAADGYLYMYNLDPQEGGECALMKQHRLDGSLETTNEILDSAS

WIPI2_HoCS    YQAG---LAVSPSRHASS---------------SA------------------LSTSNEVE--------------QNVKLSDD
WIPI2_HoCR    YQAG---LAVSPSRHASS---------------SA------------------LSTSNEVE--------------QNVKLSDD
WIPI2_Hydvu   YQAG---LAVSSSRHASC---------------SA------------------LSTSNEIE--------------QNVKISDD
WIPI2_Nemve   -----------------------------------------------------------------------------------
WIPI2_Sacko   FTKP---GGPPTFAAAAA---------------SGNRTEGTTERNVVEDYTEGLDEGTCATADLLVAARENTPTQDMLKLDDD
WIPI2_Capte   FTEPAAAAAAAPFGKTSEEGAGKGRTTEEVRKQSGNYSYASALKQAEQRAGAFFDDEPEIEVCQDDPSEVTPPQLEALRLDDE
WIPI2_Apime   GLGS---GEPPSGTTVQI---------------V-------------------QNTESEKYHEMIAATESPPGFSGSFRLKDD
WIPI2_Danre   HDRP---LVAQTYSAAVA---------------KG-----------YS-----EDQGAVGGAGVEDDM-------NTLHLDEE
WIPI2_Human   HDCP---LVTQTYGAAAG---------------KGTYVPSSPTRLAYT-----DDLGAVGGACLEDEA-------SALRLDED

WIPI2_HoCS    NEFPPLTLRNE
WIPI2_HoCR    NEFPPLTLRNE
WIPI2_Hydvu   NEFPPLTLRNE
WIPI2_Nemve   -----------
WIPI2_Sacko   NEFPPMTHDVP
WIPI2_Capte   NEFPPMTHKTD
WIPI2_Apime   AEFPPVTQRTD
WIPI2_Danre   NEQPPLILETD
WIPI2_Human   SEHPPMILRTD
```

Figure S15: Alignment of vertebrate and non-vertebrate WIPI2 protein sequences

The alignment was obtained on MUSCLE (www.ebi.ac.uk/Tools/msa/muscle/) and manually corrected to align the functional domains and the WD repeats. Accession numbers of the *Hydra* WIPI2 sequences are given in **Figure S16A**.

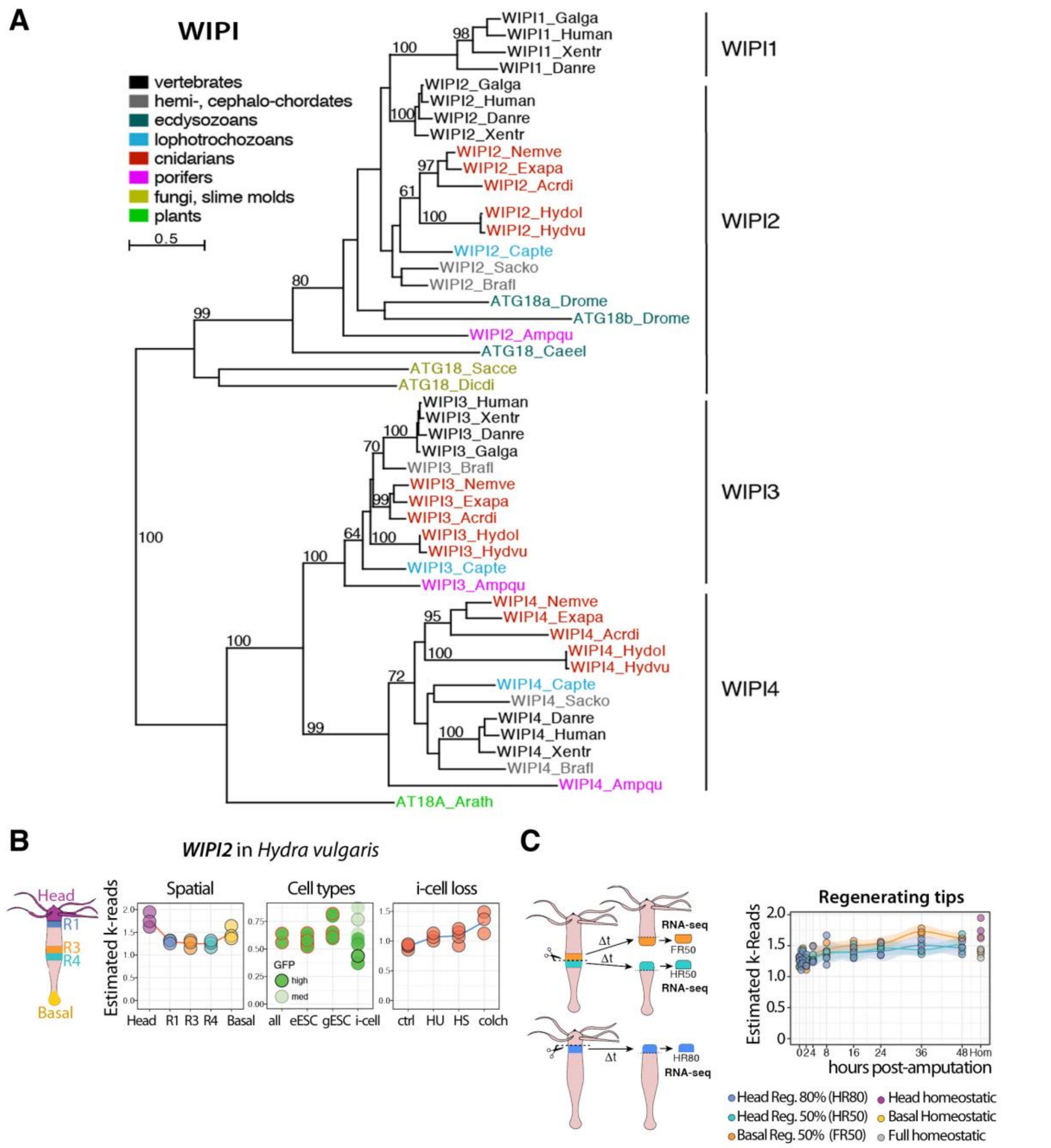

Figure S16: Phylogenetic and expression analysis of *WIPI2* in *Hydra*

(A) Phylogenetic tree of WIPI protein sequences aligned with MUSCLE and built with PhyML 3.0, tested with 100 bootstraps. Species code and sequence accession numbers: *Acrdi: Acropora digitifera* (coral, XP_015776989.1, XP_015752246.1, XP_015760656.1); *Ampqu: Amphimedon queenslandica* (XP_019853755.1, XP_003388703.1, XP_019850615.1); *Arath: Arabidopsis thaliana* (Q93VB2); *Brafl: Branchiostoma floridae* (XP_002599262.1, XP_002595393.1); *Capte: Capitella teleta* (ELT96465.1, ELU11552.1, ELT94793.1); *Danre: Danio rerio* (NP_956685.1, XP_005164182.1, Q7ZUW6, Q7ZUX3); *Dicdi: Dictyostelium discoideum* (Q54NA2); *Exapa: Exaiptasia pallida* (XP_020916524.1, XP_020900004.1, XP_020906575.1); *Galga: Gallus gallus* (XP_015135440.1, NP_001006162.1, Q5ZL16); *Human* (Q5MNZ9, Q9Y4P8, Q5MNZ6, Q9Y484); *Hydvu: Hydra vulgaris* (T2M354, T2M370, XP_012563670.1, XP_002163439.1); *Hydol* (*Ho_CS:* S022900c0g1, S037678c0g1, S028416c0g1; *Ho_CR*: R024157c0g1); *Nemve: Nematostella vectensis* (XP_001630626.1, XP_001626838.1, XP_001635768.1); *Sacce: Saccharomyces cerevisiae* (P43601); *Sacko: Saccoglossus kowalevskii* (XP_002739331.1, XP_006822202.1); *Xenla: Xenopus laevis* (Q6DCV0); *Xentr: Xenopus tropicalis* (NP_989387.1, XP_002941343.2, Q640T2). **(B, C)** RNA-seq profiles of *H. vulgaris WIPI2* as reported in (Wenger et al., 2014, Wenger et al., 2016, Wenger et al., 2019). **Body position**: expression measured at 5 distinct levels along the body axis of *H. vulgaris* Jussy strain; **Stem cell types:** expression measured in the three stem cell populations of *H. vulgaris* AEP (after FACS sorting cells of transgenic strains that constitutively GFP in one or the other cell type); **i-cell loss:** expression measured 10 days after the heat-shock or drug-induced elimination of cycling interstitial cells; **regeneration**: expression measured in regenerating tips at 9 time points of three distinct regenerative processes in *H. vulgaris* Jussy strain (HR50, FR50: head or foot regeneration after mid-gastric bisection; HR80: head regeneration after decapitation).

SUPPLEMENTARY TABLES

Interstitial lineage genes	Full protein name	UniProt AC / RefSeq *Hv*	*Ho_CR* transcript id	*Ho_CS* transcript id
CnASH	Cnidarian achaete-scute homolog	Q25179	R025980c0g2_i01	S016491c0g1_i01
Cnnos1	Cnidarian nanos-homolog 1	Q9NDP0	R036747c0g1_i01	S031114c0g1_i01
Cnnos2	Cnidarian nanos-homolog 2	Q9NDN9	R033870c0g1_i01	S025194c0g1_i01
cnox-2	Cnox-2 homeoprotein	Q9NFM1	R023828c0g2_i01	S016662c0g1_i01
COUP-TF1	COUP-TF1 nuclear orphan receptor	Q66MI8	R038175c0g3_i01	S036363c0g2_i02
foxN1	Forkhead box protein N1	T2MID9	R036487c0g1_i02	S040839c0g1_i02
foxO	FoxO transcription factor	J7HWF0	R038309c1g1_i02	S042977c0g1_i07
Hyzic	Zn-finger transcription factor 1	Q6T520	R067356c0g1_i01	S015485c0g1_i01
Kazal-1	Kazal-type serine protease inhibitor 1	Q1XEF1	R040495c0g3_i05	S040076c1g2_i07
myc1	C-Myc-binding protein 1	D0EM49	R028868c0g1_i01	S034530c0g2_i01
Notchl4	neurogenic locus notch homolog protein like 4	XP_012557050.1	R036182c1g1_i01	S041501c1g1_i01
NOWA	Nematocyst outer wall antigen	Q8IT70	R038006c0g1_i01	S038314c1g1_i01
Pax-A	Paired-box homeoprotein A	O02015	R031053c1g1_i01	S036858c0g5_i01
POU4F2	POU domain protein	T2MDR7	R026985c0g2_i02	S024242c0g1_i01
prdl-b	Paired-like homeoprotein b	O62546	R031740c0g1_i01	S030596c0g1_i01
Pumilio	Pumilio domain-containing protein KIAA0020	T2MDF1	R039094c0g1_i02	S040698c0g1_i01
RFamide-A	Neuropeptide RFamide A	O76948	R035154c0g1_i01	S036815c0g1_i01
CnVas1	Vasa-related protein CnVAS1	Q9GV13	R025460c0g1_i01	S033134c0g2_i01
CnVas2	Vasa-related protein CnVAS2	Q9GV12	R033160c0g1_i01	S042823c1g1_i02
ZNF845	Transcription factor ZNF845	I3V7W9	R003173c0g2_i01	S037612c0g1_i01

Table S1: Sequence Accession Numbers of 20 *H. vulgaris (Hv)* and *H. oligactis (Ho_CS*, *Ho_CR)* genes involved in proliferation and/or differentiation of interstitial cell (i-cell) lineages.

For the cold-induced RNA-seq profiles in *Ho_CS* and *Ho_CR*, see **supplemental Figure-S2**. For the spatial, cell-type, i-cell loss and regeneration RNA-seq profiles of the corresponding transcripts in *H. vulgaris*, see on HydrATLAS: https://HydrATLAS.unige.ch (Wenger et al., 2019).

Development • Supplementary information

Cell cycle orthologs	Full protein name	UniProt AC / RefSeq *Hv*	*Ho_CR* transcript id	*Ho_CS* transcript id
AURKA	Aurora kinase A	T2MJJ8	R027511c0g1_i04	S041489c3g2_i03
C12orf11	Cell cycle regulator Mat89Bb homolog	T2M413	R038671c0g1_i02	S041657c1g1_i02
CABLES1	CDK5 and ABL1 enzyme substrate 1	T2M990	R029958c0g1_i01	S029735c0g1_i01
CBP	CREB-binding protein	E9AI12	R039021c0g1_i04	S039796c0g2_i01
CCNA	mitotic-specific cyclin-A	P51986	R038551c0g1_i03	S039058c0g3_i03
CCNB	mitotic-specific cyclin-B	P51987	R038974c1g1_i01	S042648c3g5_i03
CCNB3	mitotic-specific cyclin-B3	T2M7Z1	R024808c0g2_i01	S036219c0g1_i01
CCND2	G1/S-specific cyclin-D2	T2MGB1	R031658c0g1_i05	S035897c0g1_i01
CCNF	Cyclin-F	T2MFV5	R033334c0g1_i01	S033757c0g1_i01
CDC123	Cell division cycle protein 123 homolog	T2MHK2	R038855c0g1_i05	S043547c1g1_i01
CDC16	Cell division cycle protein 16 homolog	T2MDN5	R021851c0g1_i01	S021110c0g1_i01
CDC20	Cell division cycle protein 20 homolog	T2MEB9	R032120c0g1_i02	S036535c0g1_i02
CDC23	Cell division cycle protein 23 homolog	T2M3J4	R036686c0g1_i04	S036439c0g1_i02
CDC27	Cell division cycle protein 27 homolog	T2MGT8	R035608c0g1_i01	S036141c0g1_i01
CDC42	Cell division control protein 42 homolog	T2MEG1	R038235c0g1_i01	S033376c0g1_i01
CDC45	Cell division control protein 45 homolog	T2MHN2	R039382c0g1_i02	S039367c0g6_i01
CDC5L	Cell division cycle 5-like protein	T2M796	R026217c0g1_i03	S038652c0g1_i03
CDC6	Cell division control protein 6 homolog	T2M680	R037857c0g1_i01	S030092c0g1_i01
CDC7	Cell division cycle 7-related protein kinase	T2MIW7	R027651c0g1_i02	S035902c0g1_i01
CDCA7L	Cell division cycle-associated 7-like protein	T2M7K7	R030222c0g1_i01	S033291c0g1_i01
DIAPH2	Protein diaphanous homolog 2	T2MIT5	R037202c0g1_i01	S041348c0g1_i02
DOT1L	Histone-lysine N-methyltransferase, H3 lysine-79 specific	T2M8S1	R033812c0g1_i01	S037123c0g1_i01
E2F4	Transcription factor E2F4	T2MCU6	R029967c0g1_i01	S008670c0g2_i01
FGFR	Fibroblast growth factor receptor	Q86PM4	R033445c0g1_i01	S034028c0g1_i01
FNTB	Protein farnesyltransferase subunit beta	T2MFI9	R007519c0g1_i01	S003373c0g1_i01
GAS2L1	GAS2-like protein 1	T2M790	R033006c0g2_i01	S034987c0g1_i01
HUS1	Checkpoint protein HUS1	T2MIV2	R011216c0g1_i01	S029062c1g1_i04
ING4	Inhibitor of growth protein	T2M3P3	R035845c0g1_i01	S001006c0g1_i01
KATNA1	Katanin p60 ATPase-containing subunit A1	T2MHM7	R033726c0g1_i01	S035284c0g1_i02
LIN52	Protein lin-52 homolog	T2MBY0	R015400c0g2_i01	S024491c0g1_i01
LIN9	Protein lin-9 homolog	T2MBY8	R032851c0g1_i01	S027133c0g1_i01
MFN2	Mitofusin-2	T2MHD7	R038385c0g1_i01	S030617c0g1_i01
MIIP	Migration and invasion-inhibitory protein	T2MC10	R033074c0g2_i01	S037583c1g1_i01
MNAT1	CDK-activating kinase assembly factor MAT1	T2MF88	R035361c2g1_i01	S042834c3g1_i02
MRE11A	Double-strand break repair protein MRE11A	T2MFZ1	R037286c0g1_i01	S039847c0g1_i02
NPDC1	Neural proliferation differentiation and control protein 1	T2M4U6	R031720c0g1_i01	S038708c0g1_i01
PA2G4	Proliferation-associated protein 2G4	T2M2R0	R038317c0g1_i01	S041220c0g1_i01
PAFAH1B1	Lissencephaly-1 homolog	T2MFT1	R036160c0g1_i01	S039362c0g1_i02
PLK1	Serine/threonine-protein kinase PLK1	T2MFR1	R038084c0g2_i01	S038822c0g1_i01
PLK4	Serine/threonine-protein kinase PLK4	T2MJ85	R031836c0g1_i01	S034548c0g2_i02
RAD1	Cell cycle checkpoint protein RAD1	T2MID6	R031826c0g1_i01	S036513c0g3_i02
RAD17	Cell cycle checkpoint protein RAD17	T2MIH3	R040845c0g1_i01	S041741c2g1_i01
RAD9A	Cell cycle checkpoint control protein RAD9A	T2M799	R040444c0g1_i02	S040626c0g1_i01
RSK	Ribosomal protein S6 kinase	E9AI11	R023522c0g2_i01	S008723c0g1_i01
SAV1	Protein salvador homolog 1	T2M622	R038127c0g1_i01	S040596c0g1_i02
SEPT2	Septin-2	T2MD65	R035337c0g1_i01	S039793c0g2_i01
SIPA1L3	Signal-induced proliferation-associated 1-like protein 3	T2MIG6	R036824c0g2_i01	S042925c0g3_i05
TFDP1	Transcription factor Dp-1	T2MDH4	R001653c0g1_i01	S030116c0g1_i02
TMEM30A	Cell cycle control protein 50A	T2M525	R031410c0g1_i01	S038468c1g1_i01
TTC28	Tetratricopeptide repeat protein 28	T2M8B7	R038479c0g1_i04	S041972c0g1_i02
TTK	Dual specificity protein kinase TTK	T2MG79	R008001c0g1_i01	S028488c0g1_i01
USPL1	Ubiquitin-specific peptidase-like protein 1	T2MBR4	R036743c0g1_i02	S040029c0g1_i06

Table S2: Sequence Accession Numbers of 52 *H. vulgaris (Hv)* and *H. oligactis (Ho_CS, Ho_CR)* orthologs to mammalian genes involved in cell cycle and cell proliferation.

For the comparative analysis of the expression of these genes after transfer to cold in *Ho_CS* and *Ho_CR*, see **Figure S3** and **Figure S4**. For the spatial, cell-type, i-cell loss and regeneration RNA-seq profiles of the corresponding transcripts in *H. vulgaris*, see on HydrATLAS: https://HydrATLAS.unige.ch (Wenger et al., 2019).

Autophagy orthologs	Full protein name	*Hv* UniProt / RefSeq	*Ho_CR* transcript id	*Ho_CS* transcript id
AMBRA1	Activating molecule in BECN1-regulated autophagy protein 1	T2M6D7	R033532c0g1_i01	S034160c0g2_i01
ATG10	Ubiquitin-like-conjugating enzyme ATG10	T2M5V2	R030322c0g1_i01	S032762c0g1_i01
ATG101	Autophagy-related protein 101	T2M6Y4	R055757c0g1_i01	S071050c0g1_i01
ATG12	Ubiquitin-like protein ATG12	T2MIE8	R029364c0g1_i01	S036036c1g1_i01
ATG13	Autophagy-related protein 13	T2MI85	R029409c0g1_i01	S043484c2g1_i01
ATG14	Beclin 1-associated autophagy-related key regulator	T2MBM0	R036673c0g1_i04	S028223c0g2_i02
ATG16L1	Autophagy-related protein 16-1	T2MC97	R036776c1g1_i01	S036117c0g1_i02
ATG2B	Autophagy-related protein 2 homolog	T2M8E3	R026665c0g1_i01	S042169c0g1_i01
ATG3	Autophagy-related protein 3	T2M4W2	R035592c1g1_i05	S040217c0g1_i08
ATG4B	Cysteine protease ATG4B	T2M2V7	R038184c0g1_i02	S037421c1g1_i01
ATG4C	Cysteine protease ATG4C	T2M7B1	R037362c0g1_i03	S043278c1g1_i01
ATG5	Autophagy protein 5	T2M5L4	R021841c0g2_i01	S030832c0g1_i01
ATG7	Ubiquitin-like modifier-activating enzyme ATG7	T2MHR4	R036000c4g1_i04	S040163c0g1_i04
ATG9A	Autophagy-related protein 9A	T2MBB7	R032526c0g4_i02	S041696c3g4_i01
BECN1	Beclin1	T2MDF4	R040450c1g2_i06	S043504c0g1_i03
CALR	Calreticulin	T2MFY9	R015676c0g2_i01	S028677c1g1_i01
CBL	E3 ubiquitin-protein ligase CBL	T2MG42	R038366c0g1_i02	S034365c0g1_i01
CDK2	Cyclin-dependent kinase 2	T2MG16	R026975c0g1_i01	S026262c0g1_i01
CLTC	Clathrin heavy chain	T2MEN5	R038444c0g1_i01	S039376c0g1_i02
CTNNB1	b-catenin	T2MGP6	R031422c0g1_i01	S035025c0g1_i01
DAPK1/MYLK1	Myosin light chain kinase	XP_012566973.1	R040005c0g1_i01	S042661c1g1_i01
DAPK2	Death-associated protein kinase 2	T2M3L1	R037170c1g1_i01	S035730c0g1_i04
DRAM1	DNA damage-regulated autophagy modulator protein 1	T2M9Y1	R010350c0g1_i01	S017218c0g1_i01
DRAM2	DNA damage-regulated autophagy modulator protein 2	T2MB87	R029953c0g1_i01	S037063c0g1_i02
DVL3	Dishevelled-like	Q9GTJ8	R035715c0g1_i01	S028887c0g3_i01
EPG5	Ectopic P granules protein 5 homolog	T2M4L4	R033380c0g1_i03	S041881c0g1_i01
FUNDC2	FUN14 domain-containing protein 2	T2M6E3	R034657c0g1_i01	S040426c0g1_i01
GABARAP	Gamma-aminobutyric acid receptor-associated protein	T2MID2	R034299c0g1_i01	S041977c0g1_i01
GABARAPL2	Gamma-aminobutyric acid receptor-associated protein like 2	T2MFA6	R040572c3g1_i01	S042989c1g3_i05
GOPC	Golgi-associated PDZ and coiled-coil motif-containing protein	T2M5L1	R025782c0g1_i01	S021196c0g1_i01
LAMP1	Lysosome-associated membrane glycoprotein 1	T2MGK4	R034674c0g2_i01	S037683c2g1_i01
LC3A/B	Microtubule-associated proteins 1A/1B light chain 3A	XP_012555909.1	R033468c0g1_i01	S043022c1g3_i01
LC3C	Microtubule-associated proteins 1A/1B light chain 3C	T2M644	R036327c0g1_i01	S040689c0g1_i01
MAPK15	Mitogen-activated protein kinase	T2M8C8	R032105c0g1_i01	S035992c0g1_i02
MFN2	Mitofusin	T2MHD7	R038385c0g1_i01	S030617c0g1_i01
mTOR	S/T protein kinase Target of Rapamycin	T2MFU7	R038760c0g1_i01	S039716c0g1_i01
MYH10	Myosin-10	T2MG36	R041168c0g3_i01	S043809c0g1_i03
NBR1	Next to BRCA1 gene 1 protein	XP_002169141.3	R036941c0g1_i01	S037290c0g1_i02
OPTN	Optineurin	T2M7C5	R030608c0g1_i01	S040493c0g1_i01
P62/SQSTM1	Sequestosome-1	T2MDZ6	R040075c0g1_i01	S041284c0g1_i03
PASK	PAS domain-containing serine/threonine-protein kinase	T2M7I6	R035698c0g1_i02	S035923c0g3_i01
PIK3R4 *(VPS15)*	Phosphoinositide 3-kinase regulatory subunit 4	T2M6A2	R032824c0g1_i01	S033802c0g1_i01
PIK3C3 *(VPS34)*	Phosphatidylinositol 3-kinase catalytic subunit type 3	T2M8P5	R028513c0g2_i01	S037049c0g1_i01
PLEKHF2	Pleckstrin homology domain-containing family F member 2	T2M5S9	R038718c0g1_i02	S041724c0g1_i01
PRKAA2	5'-AMP-activated protein kinase catalytic subunit alpha-2	T2MFI8	R036403c0g1_i02	S039556c0g1_i01
PRKAG2	5'-AMP-activated protein kinase subunit gamma-2	T2M3A1	R037932c0g1_i02	S042780c0g5_i04
PSMD4	26S proteasome non-ATPase regulatory subunit 4	T2MF29	R032102c0g1_i01	S038651c0g3_i01
RAB24	Ras-related protein Rab-24	T2M8J9	R033692c0g2_i01	S043114c1g3_i01
RB1CC1	RB1-inducible coiled-coil protein 1	T2M8Y6	R040512c0g1_i04	S040101c0g1_i01
RUBCN	Run domain Beclin-1-interacting Cys-rich domain-cont. protein	T2M8H1	R033800c0g1_i02	S036891c0g1_i01
SCOC	Short coiled-coil protein	T2M358	R027960c0g1_i01	S031987c0g1_i01
SESN1-2	Sestrin-1	T2M1Y1	R034652c0g1_i01	S030353c0g1_i01
SH3GLB1	Endophilin-B1	T2M3B1	R022339c0g1_i02	S031988c0g1_i03
STK11	Serine/threonine-protein kinase 11	T2MDA0	R028673c0g1_i01	S030765c0g1_i01
STX17	Syntaxin-17	T2MEJ3	R038040c0g1_i05	S040560c0g1_i01
TBC1D14	TBC1 domain family member 14	T2M3G3	R035499c0g1_i01	S042386c0g3_i01
TBC1D25	TBC1 domain family member 25	T2MCX7	R024677c0g1_i03	S037186c0g1_i04
TBC1D5	TBC1 domain family member 5	T2M8P8	R001078c0g2_i01	S027212c0g1_i01
TFEB (MITF)	Transcription factor EB	T2MHT1	R032064c0g1_i03	S029890c0g1_i01
TMEM192	Transmembrane protein 192	T2MAC7	R025234c0g1_i04	S029317c0g1_i01
TOLLIP	Toll-interacting protein	T2M581	R032916c0g1_i01	S064218c0g1_i01
TP53INP1	Tumor protein p53-inducible nuclear protein 1	XP_012566192.1	R036441c0g1_i01	S042256c0g3_i01
ULK1/2	Serine/threonine-protein kinase ULK1/2	XP_002167716.3	R034566c0g1_i03	S032016c0g3_i01
ULK3	Serine/threonine-protein kinase ULK3	T2MBQ7	R023417c0g1_i01	S030594c0g1_i01
ULK4	Serine/threonine-protein kinase ULK4	T2M8D5	R038809c0g1_i01	S035052c0g2_i01
USP5	Ubiquitin carboxyl-terminal hydrolase 5	T2MFQ7	R035151c0g1_i02	S036059c0g1_i03
UVRAG	UV radiation resistance-associated gene protein	T2M3F0	R011659c0g1_i01	S031929c0g1_i02
VAMP3	Vesicle-associated membrane protein 3	T2MCV8	not found	not found
VAMP4	Vesicle-associated membrane protein 4	T2MI55	R007986c0g1_i01	S030362c0g1_i01
VAMP7	Vesicle-associated membrane protein 7	T2MF92	R033884c0g3_i01	S021726c0g1_i01
VAPB	Vesicle-associated membrane protein-associated protein B/C	T2MI95	R039749c0g1_i02	S037148c1g2_i01
VMP1	Vacuole membrane protein 1	T2M837	R031782c0g1_i01	S033370c0g1_i04
VPS13A	Vacuolar protein sorting-associated protein 13A	T2M7E9	R036423c0g1_i01	S039033c0g1_i01
VPS53	Vacuolar protein sorting-associated protein 53	T2MBI0	R032923c0g1_i05	S035892c0g2_i03
WIPI2	WD repeat domain phosphoinositide-interacting protein 2	T2M354	R024157c0g1_i01	S022900c0g1_i02
ZFYVE1	Zinc finger FYVE domain-containing protein 1	T2M5M0	R008385c0g2_i01	S030332c0g1_i01

Table S3: Sequence Accession Numbers of 75 *H. vulgaris (Hv)* and *H. oligactis (Ho_CS, Ho_CR)* orthologs to the mammalian autophagy genes.

For the comparative analysis of the cold-induced gene modulations in *Ho_CR* and *Ho_CS*, see the **Figure S10** and **Figure S11**. For the spatial, cell-type, i-cell loss and regeneration RNA-seq profiles of corresponding transcripts in *H. vulgaris*, see on HydrATLAS: https://HydrATLAS.unige.ch (Wenger et al., 2019).

Gene names	Primer names	Primer sequences
**mCherry**	mCherry-for1	CAGGGGCCCCTGGGATCCCCATGGCCGATGATGAAGTTGC
	mCherry-rev1	AGTTCTTCTCCTTTACTCATTTTATATAATTCATCCATTCCACCTG
**eGFP**	eGFP-for1	TGGAATGGATGAATTATATAAAATGAGTAAAGGAGAAGAACTTTTC
	eGFP-rev1	TACTTCTGAGCCATGCATGCTTTGTATAGTTCATCCATGCCA
**hyLC3A/B**	LC3A-for1	GCTGGATGAACTATACAAAGCATGCATGGCTCAGAAGTA
	LC3A-rev1	CGCGCGAGGCAGATCGTCAGGAATTCTTAAAAATTAATGTAAGAACCAA

Table S4: Sequences of the primers used to build the mCherry-GFP-LC3A autophagy sensor

Gene names	siRNA names	siRNA sequences
p62/SQSTM1 *H. oligactis*	Ho-p62-siRNA1	CAAAGCUUCUGAAGUUUCA
	Ho-p62-siRNA2	CUCAAAUGGCUGCUAAUUA
	Ho-p62-siRNA3	AGAACAUGUUGGAGUUACU
p62/SQSTM1 *H. vulgaris*	Hv-p62-siRNA1	CAACGUUUCUGAAGUUAUA
	Hv-p62-siRNA2	UGCAAGCAAUAAUGAAGAA
	Hv-p62-siRNA3	AGCCAGCUCAAUCAAAUAA
WIPI2 *H. vulgaris*	WIPI2_siRNA1	GCAAAUGGAGCCGAUCCUU
	WIPI2_siRNA2	GCAACUAUAGCUAUCCUAA
	WIPI2_siRNA3	GGAAGAACCAAGUAGCCAA
**scrambled**	scramble-siRNA	AGGUAGUGUAAUCGCCUUG

Table S5: Sequences of the siRNA primers used to silence *p62/SQSTM1* and *WIPI2*

Targeted protein	Type	Raised in	Supplier	Ref. number	Dilution /IF	Dilution/ WB
**Ubiquitin**	monoclonal	mouse	Enzo Life Sciences	BML-PW0755-0025	1:200	NA
**Ubiquitin**	monoclonal	rabbit	Abcam	ab137025	NA	1:2000
**Human LC3B**	polyclonal	rabbit	Novus Biologicals	nb100-2220	1:300	1:1000
***Hydra* p62/SQSTM1**	polyclonal	mouse	Delphi Genetics	custom made	1:200	1:1000
**Sea urchin α-tubulin**	monoclonal	mouse	Sigma-Aldrich	T5168	1:300	NA
**Sea urchin β-tubulin**	monoclonal	mouse	Sigma-Aldrich	T5293	NA	1:2000

Table S6: List of the antibodies used in this study.

	Ho_CS						***Ho_CR***						***Hv***					
**Day post-HU**	C1	C2	C3	C4	C5	C6	C1	C2	C3	C4	C5	C6	C1	C2	C3	C4	C5	C6
**0**	10	10	10	10	10	10	10	10	10	10	10	10	10	10	10	10	10	10
**3**	10	10	10	9	10	10	10	10	10	10	10	10	10	10	10	10	10	10
**7**	10	10	10	9	10	10	10	10	10	9	10	10	10	10	10	10	10	10
**11**	10	9	7	9	10	10	10	10	10	9	10	10	10	10	10	10	10	10
**15**	10	9	7	9	10	10	10	10	10	9	10	10	10	10	10	10	10	10
**21**	10	9	7	9	10	10	10	10	10	9	10	10	10	10	10	10	10	10
**26**	8	9	5	9	9	9	9	9	10	8	9	10	10	10	10	10	10	10
**30**	8	9	4	7	9	7	6	9	8	8	7	8	10	10	10	10	10	10
**34**	5	4	2	2	5	3	1	3	3	4	2	2	10	10	10	10	10	10
**38**	2	1	1	1	4	2	0	2	1	1	0	2	10	10	10	10	10	10
**42**	1	0	1	0	1	2	0	0	0	0	0	0	10	10	10	10	10	9
**46**	0	0	0	0	0	0							9	9	9	10	10	9
**49**													9	9	9	10	8	9
**52**													9	9	8	10	8	9
**58**													8	7	7	10	8	6
**63**													6	6	6	8	7	4
**65**													5	5	5	8	6	4
**70**													3	4	4	8	5	4
**74**													3	4	4	6	5	4
**77**													3	4	4	6	4	4
**81**													3	4	4	6	3	4
**85**													2	2	0	4	3	3
**88**													1	1	0	4	3	2
**93**													1	1	0	2	3	1
**100**													0	0	0	0	0	0

Table-S7: Number of animals at different days after HU release (Figure 2I raw data). C: cohort.

	Ho_CS						***Ho_CS*** **+0.8 µM Rapamycin**					
Day at 10°C	**Cohort1**	**Cohort2**	**Cohort3**	**Cohort4**	**Cohort5**	**Cohort6**	**Cohort1**	**Cohort2**	**Cohort3**	**Cohort4**	**Cohort5**	**Cohort6**
0	10	10	10	10	10	10	10	10	10	10	10	10
10	10	10	10	10	10	10	10	10	10	10	10	10
14	10	10	10	10	10	10	10	10	10	10	10	10
18	10	10	10	10	10	10	10	10	9	10	10	10
22	10	10	10	10	10	10	10	10	9	10	10	10
28	10	10	10	10	10	10	10	10	9	10	10	10
33	9	9	9	10	8	9	10	10	9	10	10	10
37	6	9	9	8	7	9	10	10	9	10	10	10
41	2	8	6	6	5	7	10	10	9	10	10	9
45	1	7	5	5	5	6	10	10	9	10	10	9
49	1	4	5	3	5	6	10	8	9	9	10	7
53	1	4	2	2	5	3	8	7	8	8	10	7
56	1	4	1	1	2	3	7	6	8	7	10	6
59	0	4	0	1	1	2	7	4	8	7	10	4
65	0	2	0	1	1	1	7	1	7	5	7	3
70	0	2	0	1	0	1	4	0	6	5	4	2
72	0	2	0	1	0	1	4	0	6	5	4	2
77	0	2	0	0	0	1	4	0	6	5	2	1
81	0	2	0	0	0	1	4	0	6	3	2	1
84	0	2	0	0	0	1	4	0	6	3	1	1
89	0	1	0	0	0	1	3	0	6	3	1	1
93	0	0	0	0	0	0	2	0	5	2	1	1
95							1	0	4	2	1	1
100							1	0	3	1	1	0
107							1	0	3	1	0	0
113							1	0	2	0	0	0

Table-S8: Number of animals at different days after transfer to 10°C release, continuously exposed or not to rapamycin (0.8 µM) (Figure 6B raw data).

SUPPLEMENTARY MOVIES

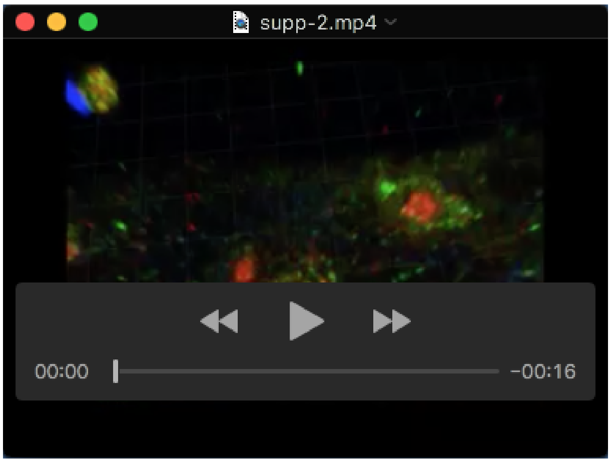

Movie 1: 3D-reconstruction of LC3 decorated p62/SQSTM1 bodies

LC3 decorated p62/SQSTM1 bodies identified in epithelial cells of *Ho_CS* polyps macerated after 35 days at 10°C. Image acquired on a Leica SP8 confocal microscope, 3D reconstruction performed with Bitplane Imaris.

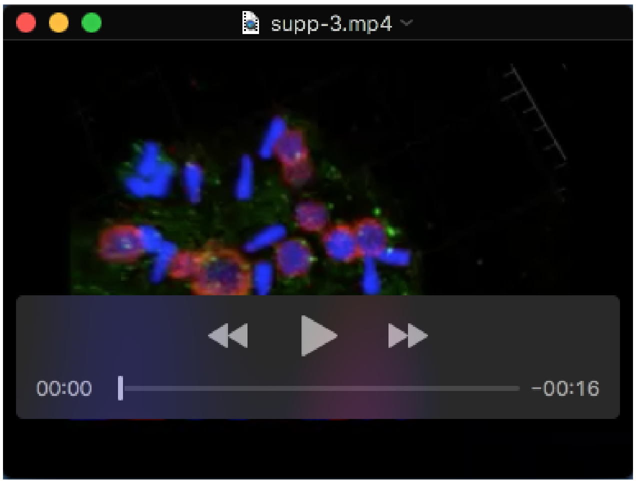

Movie 2: 3D-reconstruction of an epithelial cell having engulfed germ cells identified in 35 days old *Ho_CS* polyps treated with Rapamycin

3D reconstruction with Bitplane Imaris of the confocal image of engulfed germ cells decorated with p62/SQSTM1 or p62/SQSTM1-LC3 in e-cells of *Ho_CS* polyp maintained at 10°C for 35 days and continuously treated with Rapamycin.

Development • Supplementary information

SUPPLEMENTARY REFERENCES

Birgisdottir, A. B., Lamark, T. and Johansen, T. (2013). The LIR motif - crucial for selective autophagy. *J Cell Sci,* **126,** 3237-47.

Bitto, A., Lerner, C. A., Nacarelli, T., Crowe, E., Torres, C. and Sell, C. (2014). P62/SQSTM1 at the interface of aging, autophagy, and disease. *Age (Dordr),* **36,** 9626.

Pankiv, S., Clausen, T. H., Lamark, T., Brech, A., Bruun, J. A., Outzen, H., Overvatn, A., Bjorkoy, G. and Johansen, T. (2007). p62/SQSTM1 binds directly to Atg8/LC3 to facilitate degradation of ubiquitinated protein aggregates by autophagy. *J Biol Chem,* **282,** 24131-45.

Seibenhener, M. L., Babu, J. R., Geetha, T., Wong, H. C., Krishna, N. R., Wooten, M. W. (2004). Sequestosome1/p62 is a polyubiquitin chain binding protein involved in ubiquitin proteasome degradation. *Mol Cell Biol*, **24**, 8055-8068. doi: 10.1128/MCB.24.18.8055–8068.2004

Wenger, Y., Buzgariu, W. and Galliot, B. (2016). Loss of neurogenesis in Hydra leads to compensatory regulation of neurogenic and neurotransmission genes in epithelial cells. *Philos Trans R Soc Lond B Biol Sci,* **371,** 20150040.

Wenger, Y., Buzgariu, W., Reiter, S. and Galliot, B. (2014). Injury-induced immune responses in Hydra. *Semin Immunol,* **26,** 277-294.

Wenger, Y., Buzgariu, W., Perruchoud, C., Loichot, G., Galliot, B. (2019). Generic and context-dependent gene modulations during Hydra whole body regeneration. *BioRxiv* 587147, doi.org: 10.1101/587147

Development • Supplementary information
